# DCC-2036 induces repolarization of TAMs to M1 type and enhances CD8^+^ T cell immunity in TNBC

**DOI:** 10.1016/j.ymthe.2025.10.042

**Published:** 2025-10-24

**Authors:** Yuxin Liang, Qiting Zeng, Maoyu Xiao, Pei Li, Rongfang He, Zhangjie Chen, Jun Liu, Jingsong Cao, Jun Li, Liyang Yin, Jing Zhong, Xisha Chen, Jianbo Feng, Jun He, Xiguang Chen, Xuyu Zu, Yingying Shen

**Affiliations:** 1 Cancer Research Institute, Hunan Provincial Clinical Medical Research Center for Drug Evaluation of Major Chronic Diseases, The First Affiliated Hospital, Hengyang Medical School, University of South China, Hengyang, Hunan 421001, China; 2 Department of Clinical Laboratory Medicine, Institution of Microbiology and Infectious Diseases, Hunan Province Clinical Research Center for Accurate Diagnosis and Treatment of High-incidence Sexually Transmitted Diseases, The First Affiliated Hospital, Hengyang Medical School, University of South China, Hengyang, Hunan 421001, China; 3 Department of Pathology, The First Affiliated Hospital, Hengyang Medical School, University of South China, Hengyang, Hunan 421001, China; 4 Department of Spine Surgery, The Nanhua Affiliated Hospital, Hengyang Medical School, University of South China, Hengyang 421002, China

**Keywords:** triple-negative breast cancer, TNBC, DCC-2036, hematopoietic cell kinase, HCK, tumor-associated macrophages, TAMs, repolarization

## Abstract

Therapies for triple-negative breast cancer (TNBC) still need innovative approaches, while repolarizing tumor-associated macrophages (TAMs) may offer a breakthrough in the targeted therapy and immunotherapy of TNBC. In this study, our group found that the small-molecule tyrosine kinase inhibitor DCC-2036 could induce repolarization of TAMs from M2 to M1 type and enhance anti-tumor CD8^+^ T cell immunity in TNBC. Mechanistically, targeting inhibition of the non-receptor tyrosine kinase hematopoietic cell kinase (HCK) in TAMs regulated the downstream phosphatidylinositol 3-kinase (PI3K)/protein kinase B (AKT)-mammalian target of rapamycin (mTOR)-glutamine synthetase (GS)-HIF1α signaling pathway, leading to a reprogramming of TAM metabolism from oxidative phosphorylation to glycolysis. This metabolic shift repolarized TAMs to the M1 phenotype, resulting in a decrease in interleukin (IL)-10 secretion, which enhanced the immune response of anti-tumor CD8^+^ T cells and increased the sensitivity of TNBC to immune checkpoint blockade therapy. This project uncovers a previously unrecognized anti-tumor mechanism of DCC-2036 and proposes a combination strategy that utilizes DCC-2036 alongside immune checkpoint inhibitors to improve TNBC immunotherapy.

## Graphical abstract

**Figure:**
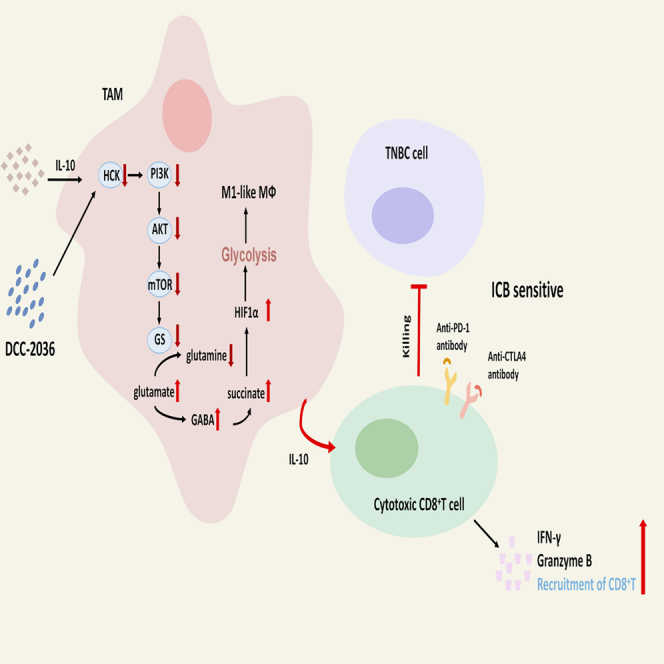

## Teaser

DCC-2036 targets the HCK-AKT/mTOR-GS-HIF1α axis in TNBC to modulate the metabolic reprogramming of TAMs, leading to repolarization of TAMs toward the M1 phenotype, resulting in a decrease in IL-10 secretion, which enhances the immune response of anti-tumor CD8^+^ T cells and increases the sensitivity of TNBC to immune checkpoint blockade therapy.

## Introduction

Breast cancer has replaced lung cancer, becoming the cancer with the highest incidence rate in the world. In China, the incidence of breast cancer has remained persistently number one among Chinese women.^1^^,^^2^ Triple-negative breast cancer (TNBC) constitutes about 10%–20% of all breast cancer cases and has the worst prognostic outcome.^3^ Since TNBC does not express estrogen receptor (ER), progesterone receptor (PR), and HER2, the patients cannot benefit from endocrine and targeted therapies, and chemotherapy remains a standard regimen. However, the chemotherapy easily develops drug resistance and severe toxic effects.^4^^,^^5^ In recent years, with the rapid development of tumor immunotherapy, immune checkpoint blockade (ICB) has shown notable efficacy in treating TNBC. Unfortunately, only a minority of patients benefit from ICB. The resistance to ICB therapy of patients with TNBC remains a great clinical challenge. The resistance to ICB is multifaceted and complex, with one of the key factors being the inhibitory tumor microenvironment (TME) (infiltration of tumor-associated macrophages [TAMs] and decreased infiltration of CD8^+^ T cells/reduced cytotoxic activity).^6^^,^^7^^,^^8^^,^^9^ At present, the treatment of patients with TNBC remains a major clinical challenge. Accordingly, the search for new therapeutic drugs and novel therapeutic targets is urgently needed.

TAMs are the major immune cells that compose the TME.^10^ The M1 macrophages (classically activated macrophages) drive anti-tumor responses and pro-inflammatory actions. Conversely, the M2 macrophages exert pro-tumorigenic and anti-inflammatory effects, and the alternatively activated M2 macrophages are polarized by cytokines such as interleukin (IL)-4, IL-10, and IL-13. TAMs tend to show the M2-like phenotype.^11^^,^^12^ Studies have increasingly shown that TAMs play a crucial role in tumor progression by regulating tumor immunosuppression, remodeling tumor extracellular matrix, and promoting angiogenesis.^13^^,^^14^ TAMs are the main immune cells in the TME of TNBC and are associated with poor prognosis and survival in TNBC.^15^ Thus, targeting TAMs presents a promising strategy for the immunotherapy of TNBC. Under specific conditions, macrophages undergo polarization toward the M1 type, effectively improving the immunosuppressive TME and enhancing anti-tumor adaptive immune responses.^16^ Several studies have indicated that metabolic reprogramming is intricately linked to the polarization of macrophages. Studies have confirmed that inhibiting the activity of glutamine synthetase (GS) in M2 macrophages alters their polarization toward an HIF1α-mediated M1 state, and this crucial process involves a shift from oxidative phosphorylation to aerobic glycolysis.^17^^,^^18^

Over the past years, tyrosine kinase inhibitors (TKIs) have emerged as important pharmacological agents in the area of targeted therapy against various cancers.^19^ Of particular concern is the emerging critical role played by TKIs in cancer immunotherapy. According to the latest systematic review and search of clinical trial registries (https://clinicaltrials.gov/), with respect to TNBC, approximately 60 TKI clinical trials have been registered. Among these, the drug tivantinib, which targets MET, is currently in a phase II clinical trial.^20^ If all breast cancer subtypes are included, the total number of TKI-related clinical trials exceeds 100 and is still increasing at a rate of five to eight trials per year. A study has discovered gefitinib inhibits the M2-like polarization of TAMs in Lewis lung cancer xenografts by targeting the STAT6 signaling pathway.^21^ DCC-2026, a third-generation TKI, is a novel small-molecule TKI with a wide spectrum of kinase inhibition.^22^^,^^23^ In a phase Ib/2 clinical study, DCC-2036 was combined with paclitaxel, which is effective in patients with platinum-resistant ovarian cancer. Our research group has found that DCC-2036 can target AXL/MET and regulate phosphatidylinositol 3-kinase (PI3K)/protein kinase B (Akt)-nuclear factor (NF)-κB signaling to inhibit TNBC cell proliferation, migration, and invasion both *in vivo* and *in vitro*.^24^ Our group has also found that DCC-2036 can inhibit TNBC stem cells by targeting the AXL-KLF5 positive-feedback loop, leading to increased chemosensitivity in TNBC cells.^25^ Studies reported that DCC-2036 suppresses luminal breast cancer and pancreatic neuroendocrine tumors (NETs) by blocking Tie2^Hi^ macrophage recruitment and function, indicating it has immunomodulatory functions.^26^ However, the influence and mechanism of DCC-2036 on tumor immunity of TNBC has not been explored.

In this study, we evaluated the influence of DCC-2036 on tumor immunity in TNBC. We have confirmed that DCC-2036 definitively acts as an immune activator in TNBC by inducing repolarization of TAMs to M1 type and enhancing anti-tumor CD8^+^ T cell immunity. Mechanistically, DCC-2036 regulates metabolic reprogramming of TAMs by targeting the hematopoietic cell kinase (HCK)-GS-HIF1α axis in TNBC.

## Results

### *In vitro* induction and characterization of M2 macrophage polarization models

Currently, mannose receptor (CD206), arginase (ARG), scavenger receptor (CD163), chemokine 5 (CCL5), and vascular endothelial growth factor (VEGF) are recognized as polarization markers associated with M2 macrophages, while inducible nitric oxide synthase (iNOS), IL-6, and IL-23 serve as polarization indicators for M1 macrophages. Research findings have demonstrated that M0 macrophages can undergo polarization into M2 phenotype upon stimulation with IL-10,^27^ and characterization of these cells relies on the assessment of expression levels of polarization markers. The qPCR experiments revealed that the induction of IL-10 in bone marrow-derived macrophages (BMDMs) resulted in an increase in mRNA expression levels of M2 markers ARG and CCL5, while a decrease was observed in the mRNA expression levels of M1 markers IL-23 and iNOS (Figure S1A). Similar findings were obtained when RAW264.7 cells were induced with IL-10 (Figure S1B). Furthermore, western blotting (WB) analysis demonstrated significantly elevated expression of CD206 and ARG in both BMDM and RAW264.7 cells after 48 h of IL-10 induction, compared to macrophages without IL-10 induction. Conversely, the expression of iNOS was lower after 48 h of IL-10 induction (Figure S1C). In addition, flow cytometry was employed to assess the expression of CD206 in BMDM and RAW264.7 cells both under normal conditions and after 48 h of IL-10 induction. It was observed that the mean fluorescence intensity of CD206 in both BMDM cells and RAW264.7 cells induced with IL-10 exhibited a significantly higher level compared to the control group (Figure S1D). According to the aforementioned experimental results, it has been demonstrated that both BMDM and RAW264.7 macrophages could undergo polarization into M2-type macrophages following a 48-h induction of IL-10, thereby indicating successful establishment of the M2 macrophage polarization models, which also could be used in the following research as TAMs *in vitro* for the functional similarity.

### DCC-2036 induces the repolarization of TAMs into M1 type and its effect on the migration and invasion of TNBC cells

**Figure 1:**
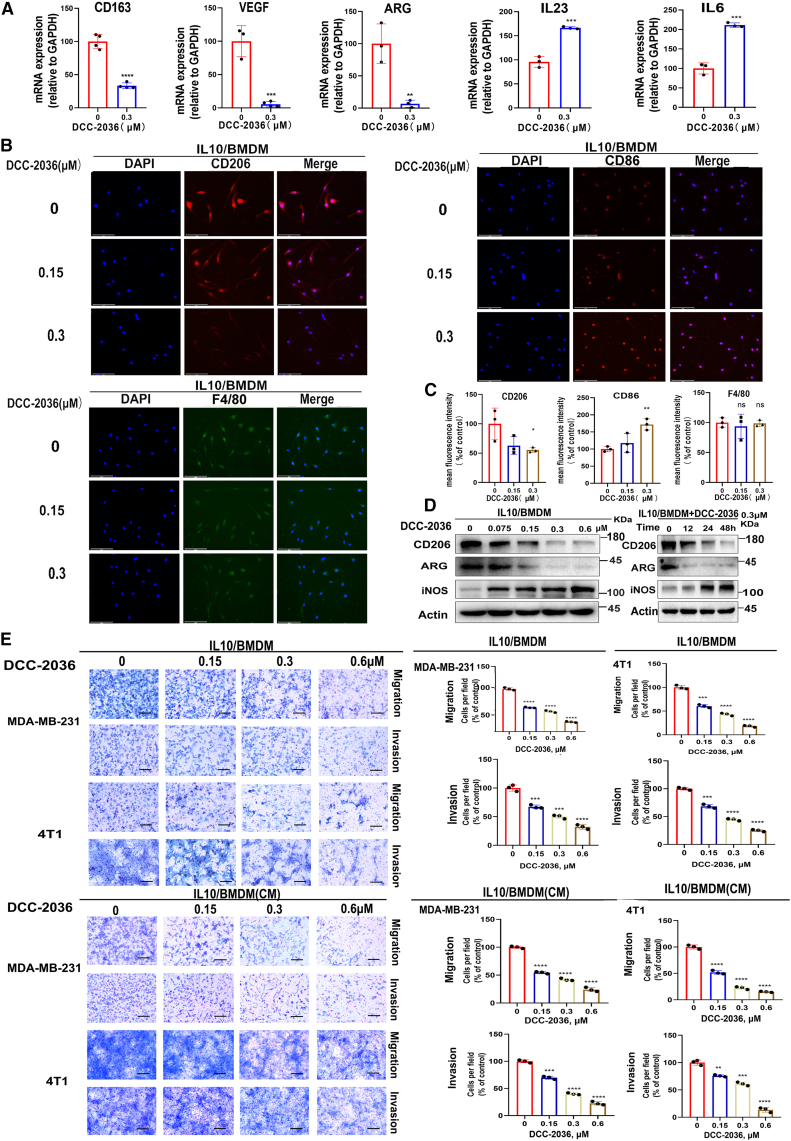
Effect of DCC-2036 on the polarization of the M2-type macrophages, IL-10/BMDM, and its effect on the migration and invasion of TNBC cells IL-10/BMDMs were treated with DCC-2036 with indicated concentrations for 48 h. (A) Expression of M1 and M2 macrophage markers in M2-type macrophages treated with DCC-2036 (at a concentration of 0.3 μM) *in vitro* was detected by qPCR. (B) Immunofluorescence detection of mean fluorescence intensity of M1 and M2 markers in IL-10/BMDMs treated with different concentrations of DCC-2036. Scale bars, 75 μm. (C) Former results are shown in bar charts (*n* = 3 independent experiments). (D) Different concentrations of DCC-2036 were added in IL-10/BMDM cells for 48 h; DCC-2036 at a concentration of 0.3 μM was applied to IL-10/BMDMs in a time gradient, and cells were collected for WB to analyze protein expression of M1 and M2 macrophage markers. (E) IL-10/BMDMs treated with different concentrations of DCC-2036 were co-cultured with MDA-MB-231 and 4T1 cells; conditioned medium (CM) of IL-10/BMDM cells treated with different concentrations of DCC-2036 was co-cultured with MDA-MB-231 and 4T1 cells. Scale bars, 50 μm. The statistical significance was determined by Student’s t test: ∗*p* < 0.05,∗∗*p* < 0.01,∗∗∗*p* < 0.001,∗∗∗∗*p* < 0.0001.

Subsequently, we examined the impact of the small-molecule TKI DCC-2036 on the repolarization of TAMs (M2 macrophages) within an IL-10-induced polarization model. BMDMs derived from mouse bone marrow were treated with DCC-2036 in the presence of IL-10. The expression levels of M1 and M2 polarization markers in the treated M2 BMDMs were assessed using qPCR, immunofluorescence, and WB analyses. The qPCR results demonstrated a significant downregulation of M2 polarization markers CD163, VEGF, and ARG in response to DCC-2036 treatment in M2 BMDMs, accompanied by an upregulation of M1 markers IL-23 and IL-6 (Figure 1A). The immunofluorescence results also demonstrated a reduction in the mean fluorescence intensity of M2 marker CD206 but an elevation in the mean fluorescence intensity of M1 marker CD86 (Figures 1B and 1C). Moreover, WB results revealed a concentration-dependent and time-dependent inhibition of ARG and CD206 protein expression in the M2 BMDMs upon treatment with DCC-2036 while concurrently promoting iNOS protein expression (Figure 1D). The above findings demonstrated that DCC-2036 induced the repolarization of TAMs into M1 type. This observation has also been corroborated in the M2 RAW264.7 macrophage cells (Figures S2A–S2D). TAMs are similar to an M2-like phenotype and actively contribute to tumor progression by facilitating invasion and metastasis. Based on our above findings, we investigated whether DCC-2036 could modulate the migratory and invasive potential of TNBC cells by inducing repolarization of M2 macrophages. To assess the migratory and invasive properties of TNBC cells (MDA-MB-231 and 4T1), the Transwell assay was performed using conditioned medium (CM) obtained from DCC-2036-treated M2 BMDMs or DCC-2036-treated BMDMs co-cultured with TNBC cells. The results showed that both the CM derived from M2 BMDMs treated with DCC-2036 and DCC-2036-treated BMDMs significantly inhibited the migration and invasion of TNBC cells. Moreover, as the concentration of DCC-2036 increased, their ability to suppress the migration and invasion of TNBC cells became stronger (Figure 1E). This was also confirmed in M2 RAW264.7 cells (Figure S2E).

### DCC-2036 effectively suppresses the *in vivo* growth and metastasis of TNBC by inducing repolarization of TAMs into M1 type

**Figure 2:**
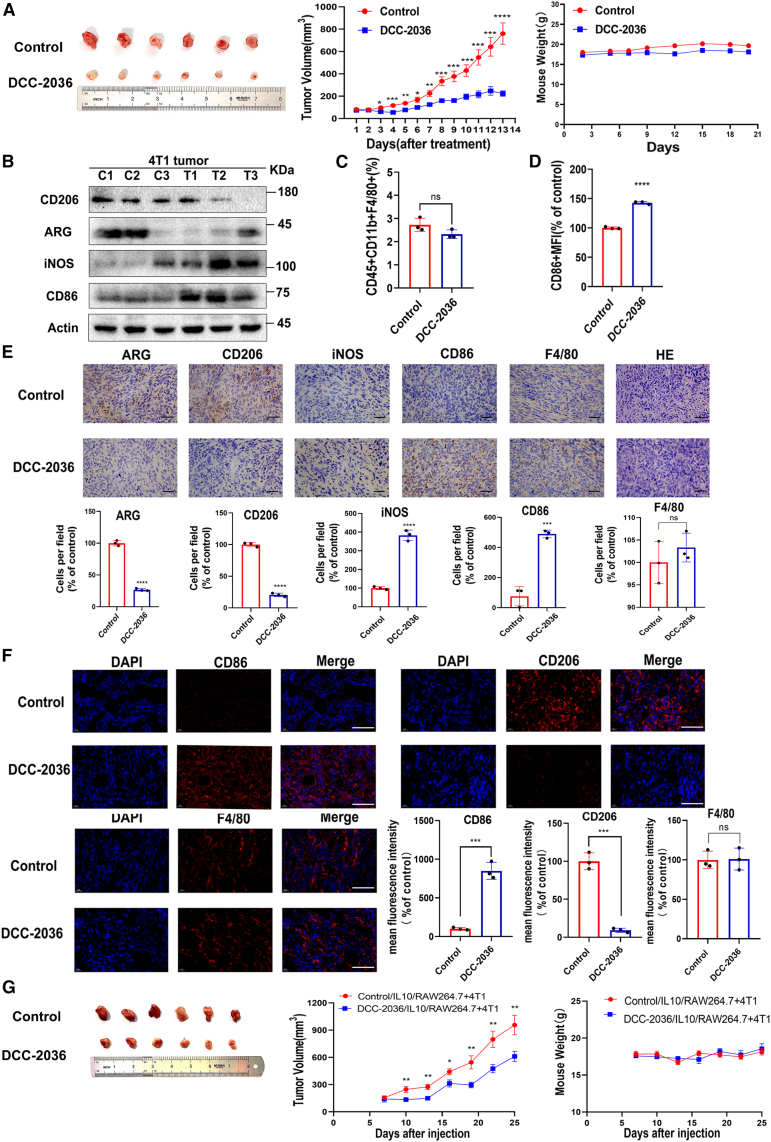
DCC-2036 induces the polarization of TAMs to inhibit the growth of TNBC *in vivo* To establish an *in situ* tumor model of TNBC, 2 × 10^6^ 4T1 cells were transplanted into the fourth pair of subcutaneous mammary fat pads of Balb/c mice. The control group (*n* = 10) was administered with a solvent control solution (0.5% CMC/1% Tween 80) via gavage, while the treatment group (*n* = 8) received oral administration of DCC-2036 at a dosage of 100 mg/kg every other day. We measured the weight of mice and dimensions of tumors on the subsequent day. (A) The representative diagram illustrates the tumor tissue size in the control group and treatment group of mice (left); tumor growth curve of control and treated mice (middle); plot of body-weight changes in control and treated mice (right). (B) We randomly selected any three tumor tissues from the control group and treatment group mice for WB analysis. (C) Flow cytometry was employed to quantify the total population of macrophages in tumor tissues from both control and treatment groups of mice. (D) Flow cytometry was employed to assess the fluorescence intensity of CD86 expression in tumor tissues from both control and treatment groups of mice. (E) IHC was used to assess the expression of ARG, CD206, iNOS, CD86, and F4/80. H&E staining indicates the histology of tumor tissues. Scale bars, 20 μm. (F) Immunofluorescence technique was employed to assess the expression of CD86, CD206, and F4/80. Scale bars,100 μm. The statistical significance was determined by Student’s t test: ∗*p* < 0.05,∗∗*p* < 0.01,∗∗∗*p* < 0.001,∗∗∗∗*p* < 0.0001. (G) The representative images compare tumor tissues from the control group (IL-10/RAW264.7+4T1, top) and treatment group (2.5 μM DCC-2036/IL-10/RAW264.7+4T1, bottom) mice (left); tumor growth kinetics in the control and treatment groups of mice (middle); weight dynamics in the control and treatment groups of mice (right). The statistical significance was determined by Student’s t test: ∗*p* < 0.05,∗∗*p* < 0.01,∗∗∗*p* < 0.001,∗∗∗∗*p* < 0.0001.

To establish an *in situ* mouse model of breast tumor, TNBC 4T1 cells were implanted into the subcutaneous fat pad of the fourth mammary gland pair in Balb/c mice. The mice were randomly divided into a control group and a treatment group. Once the average tumor volume reached 80–100 mm^3^, the mice in the treatment group received oral administration of DCC-2036 at a dosage of 100 mg/kg every other day. The tumor growth in the two groups of mice was monitored, and the subsequent analysis revealed that the treatment group exhibited a slower tumor growth rate compared to the control group, while no significant difference in weight change between the two groups was observed (Figure 2A). These findings demonstrated the inhibitory activity of DCC-2036 on the growth of TNBC cells in mice. However, further validation is required to ascertain its potential for inducing repolarization of TAMs. The tumor tissues of mice in the treatment group and control group were examined using WB (Figure 2B), immunohistochemistry (IHC) (Figure 2E), and immunofluorescence (Figure 2F) experiments. It was observed that M2 polarization markers CD206 and ARG exhibited high expression in the tumor tissues of control-group mice, whereas their expression decreased in the treatment group. Conversely, M1 polarization markers CD86 and iNOS showed low expression in the tumor tissues of control-group mice but increased expression in the treatment group. Additionally, flow cytometry analysis demonstrated that DCC-2036-treated mice displayed elevated levels of CD86 on macrophages within their tumor tissues compared to the control group. Notably, no significant difference was observed in total macrophage marker F4/80 between the tumor tissues of both groups (Figures 2C and 2D). These findings suggested that DCC-2036 could induce repolarization of TAMs into M1-type macrophages *in vivo*. To further validate the direct inhibitory effect of DCC-2036 on TNBC growth by inducing repolarization of TAMs within tumor tissues *in vivo*, we established an *in situ* co-implantation model consisting of M2 macrophages and 4T1 cells in mice. In this study, M2 macrophages (IL-10/RAW264.7), either treated or untreated with DCC-2036, were co-implanted with 4T1 cells and subsequently injected into the subcutaneous fat pad of the fourth mammary gland pair in mice to assess tumor growth. The findings revealed that tumors formed by DCC-2036-treated M2 macrophages exhibited a significantly reduced growth rate and smaller volume compared to those formed by untreated M2 macrophages and 4T1 cells. Moreover, there was no significant disparity observed in terms of body-weight changes between the two groups of mice (Figure 2G). Therefore, DCC-2036 effectively inhibited TNBC growth *in vivo* through inducing repolarization of TAMs. Simultaneously, we established a mouse model of lung metastasis in Balb/c mice through intravenous injection of 4T1-luc stable transfected cells. The mice were randomly allocated into a control group and a DCC-2036 drug treatment group. Commencing from the first day of tumor implantation, an oral gavage of 100 mg/kg DCC-2036 was administered every other day until the completion of the experiment on the 14th day. The *in vivo* IVIS imaging results demonstrated a significant attenuation of lung metastasis signal in the DCC-2036-treated group compared to the control group (Figure S3A). In panoramic scanning images of H&E-stained lung tissue sections, it was observed that the control group of three mice exhibited a total of 19 lung nodules, whereas the DCC-2036 treatment group showed only seven. Notably, compared to the control group, the DCC-2036 treatment demonstrated significant inhibition of lung metastasis in TNBC mouse models (Figure S3B). Subsequently, we conducted IHC experiments to investigate the alterations in F4/80, ARG, and CD86 (macrophage-related markers) within mouse lung nodules from the two groups. The graph revealed no significant alteration in the expression of F4/80, a macrophage marker. However, treatment with DCC-2036 significantly diminished the levels of M2-type marker ARG in lung nodules of mice and increased the expression of M1-type marker CD86 (Figure S3C). These findings suggested that DCC-2036 effectively inhibited lung metastasis in mice with TNBC by inducing polarization of TAMs from the M2 phenotype to the M1 phenotype.

### The anti-tumor effect mediated by DCC-2036 is dependent on CD8^+^ T cells

**Figure 3:**
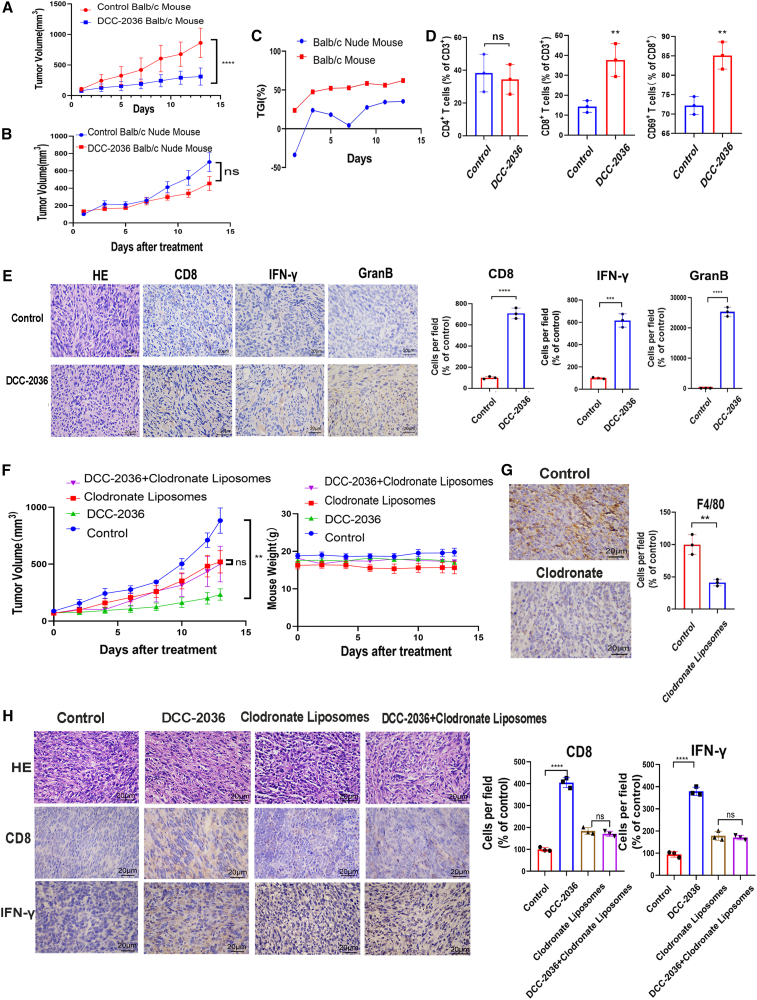
DCC-2036 enhances CD8^+^ T cell-mediated anti-tumor immunity, and this effect is dependent on the action of DCC-2036 on macrophages (A and B) To establish a TNBC *in situ* breast tumor model, 2 × 10^6^ 4T1 cells were inoculated into the subcutaneous fat pad of Balb/c mice and Balb/c nude mice. The control group received oral administration of solvent control (0.5% CMC/1% Tween 80), while the treatment group was orally administered with DCC-2036 at a dosage of 100 mg/kg every other day. Mouse body weight and tumor volume were recorded bi-daily, and a tumor growth curve was plotted based on the collected data. (C) We revealed the tumor growth inhibition (TGI) rate by analyzing tumor data. (D) Subcutaneous grafts from Balb/c mice were obtained and processed using the gentleMACS automated tissue dissociator to obtain single-cell suspensions (randomly selecting three vs. three). Flow cytometry was employed to compare alterations in CD4^+^ and CD8^+^ T cells within tumor tissues between the control group and mice treated with DCC-2036. (E) Subcutaneous transplantation of tumors in Balb/c mice was performed to conduct immunohistochemical experiments. The comparison between the control group and the DCC-2036-treated group involved assessing the number of CD8^+^ T cells (CD8) and expression levels of activation markers (IFN-γ and GranB) in tumor tissues. H&E staining indicates the histology of tumor tissues. (F) The control group (*n* = 4) was administered solvent every other day. The clodronate liposome group (*n* = 4) received a dose of 150 μL/each/time via intraperitoneal injection 1 day before and on the twelfth day after tumor implantation. The DCC-2036 group (*n* = 4) was orally gavaged with a dose of 100 mg/kg every other day. Lastly, the clodronate liposomes + DCC-2036 group (*n* = 4) received both treatments at the same doses as the previous two groups. Tumor growth and changes in body weight were monitored for all four groups. (G) IHC experiments were performed to assess the expression of total macrophage marker F4/80 following treatment with clodronate liposomes. (H) IHC experiments were performed to assess alterations in CD8^+^ T cell biomarkers within tumor tissues across four groups of mice.

One of the main functional changes caused by the phenotypic changes of TAMs is the T cell immune response, the most prominent of which is the change of CD8^+^ T cell response caused by the change of cytokines (such as IL-10 and TGF-β) secreted by TAMs.^28^ In order to investigate whether the inhibitory effect of DCC-2036 on TNBC is dependent on the activation of the T cell-mediated anti-tumor immune response, we established an *in situ* TNBC model. We compared the tumor growth curves (Figures 3A and 3B) and monitored body-weight changes (Figure S4A) in Balb/c mice treated with DCC-2036 as well as Balb/c nude mice. Additionally, we plotted the curve for tumor inhibition rate (Figure 3C). Our results demonstrated that DCC-2036 exhibited a significantly greater inhibitory effect on mouse tumors in immunocompetent Balb/c mouse models compared to T cell-deficient Balb/c nude mouse models when administered orally at a dosage of 100 mg/kg every other day. In the Balb/c mouse model, we performed flow cytometry experiments on tumor tissues from both the treatment and control groups. Our results revealed no significant alterations in the number of CD4^+^ T cells within tumor tissues between the two groups. However, a notable enhancement in both quantity and activity of CD8^+^ T cells was observed in the treatment group compared to the control group, exhibiting statistically significant differences (Figure 3D). Immunohistochemical experiments revealed a significant upregulation of CD8, interferon (IFN)-γ, and granzyme B expression (indicating T cell activation/toxicity markers) in tumor tissues of DCC-2036-treated mice compared to the control group (Figure 3E). Similarly, immunofluorescence techniques demonstrated a substantial increase in both CD8 and CD69 in the treatment group (Figure S4B). Moreover, the results of IHC experiments demonstrated a significant increase in CD8 and IFN-γ within lung nodules from the DCC-2036-treated group (Figure S4C). These findings suggested that the inhibition of TNBC by DCC-2036 was associated with enhanced activation of CD8^+^ T cell-mediated immune response. To investigate whether the activation of anti-tumor CD8^+^ T cell immune response depended on the impact of DCC-2036 on macrophages, we established an *in situ* TNBC model in mice using clodronate liposomes (successful elimination of macrophages was confirmed by F4/80 IHC staining in Figure 3G). Treatment with DCC-2036 alone significantly inhibited tumor growth compared to the control group, accompanied by increased expression levels of CD8 and IFN-γ in mouse tumor tissues. However, when clodronate liposomes were used for macrophage depletion, there was no additional improvement observed in tumor growth inhibition within the combination-therapy group (clodronate liposomes + DCC-2036) compared to the clodronate-liposome group. Moreover, this combination therapy did not perform as effectively as DCC-2036 monotherapy. The expression levels of CD8 and IFN-γ were similar between the clodronate-liposome group and the combination-therapy group (Figures 3F and 3H). These findings suggested that DCC-2036 also induced functional alterations of TAMs by enhancing anti-tumor immunity mediated by CD8^+^ T cells through its effect on macrophages. More importantly, in order to assess the direct impact of DCC-2036 on CD8^+^ T cells, we conducted an *in vitro* experiment comparing the proliferation rates and activity of CD8^+^ T cells before and after treatment with DCC-2036. Interestingly, our findings revealed comparable results (Figure S4D), suggesting that the observed augmentation in both quantity and functionality of CD8^+^ T cells induced by DCC-2036 was not attributed to its direct influence on CD8^+^ T cells.

### ADMET analysis of DCC-2036

In order to gain a deeper understanding of the characteristics of DCC-2036, we conducted an absorption, distribution, metabolism, excretion, and toxicity (ADMET) analysis. DCC-2036 exhibited favorable drug-like properties, including compliance with Lipinski and Pfizer rules, optimal TPSA (123 Å^2^) and logP (4.49), moderate molecular flexibility, and a high plasma protein-binding rate (98.8%) with a distribution volume of 1.93 L/kg, suggesting sustained peripheral exposure without significant CNS penetration. ADMET predictions indicated strong P-gp inhibition, moderate oral bioavailability (F50% +++), and controlled plasma clearance (2.14 mL/min/kg; T_1_/_2_ = 1.36 h), collectively supporting acceptable absorption, distribution, and elimination profiles (Table S2). CYPstrate analysis identified DCC-2036 as a substrate primarily of CYP2C19 and CYP3A4 but a non-substrate for other major CYP isoforms, indicating reduced risk of multi-enzyme-mediated clearance and drug-drug interactions (Table S3). SULT predictions demonstrated non-substrate status across all major SULT family members, consistent with the absence of –OH or –NH_2_ groups, implying minimal SULT-mediated clearance and low formation of reactive sulfated metabolites (Table S4). GLORYx metabolic mapping identified primary reactions as N-oxidation, aromatic hydroxylation, N-acetylation, and selective hydrolysis, with high-priority scores, while sulfation pathways were absent, reinforcing metabolic stability (Table S5). Collectively, these features—controlled metabolism, low risk of reactive metabolite formation, favorable ADMET profile, and selective enzyme interactions—support the suitability of DCC-2036 as a promising drug candidate.

### The non-receptor tyrosine kinase HCK emerges as a pivotal target for the therapeutic efficacy of DCC-2036 in TAMs

DCC-2036 was originally designed as a multi-target inhibitor based on the ABL1 structure.^22^ To identify the key target of DCC-2036 in TAMs, we first screened for ABL1 homologs. By removing compounds from the crystal structure of the ABL1 inhibitor DCC-2036 and using it as a template, we modeled each kinase domain using the Modeller program, ensuring that all kinase domains could accommodate binding of DCC-2036. We reproduced the binding of ABL1 with DCC-2036 using Autodock to validate the docking method. Subsequently, DCC-2036 was docked to each kinase domain using the same method, and the result with the lowest binding energy was selected as the final model. As shown in the sequence alignment result (Figure S5A), the ABL1 homologs include BTK, CSK, FGR, FLT3, FYN, HCK, KIT, LCK, LYN, SRC, TIE2, and YES. These protein kinase domains all have structural elements similar to ABL1 (Figure S5B). To demonstrate the binding differences between DCC-2036 and different kinases, we performed molecular docking using homology models. The results showed that ABL1 had the highest affinity for DCC-2036 (−13.7 kcal/mol), while DCC-2036 had a binding free energy of −12.2 kcal/mol with HCK, ranking relatively high among ABL1 homologs (Table S6). Notably, DCC-2036 binds to HCK in the type II model, which is the DFG-out conformation. In this complex, HCK residues Met336, Phe335, Thr333, Asp398, Met309, Lys290, Tyr376, and Glu305 form hydrogen bonds with various functional groups of DCC-2036, in agreement with the previously reported ABL1/DCC-2036 structure (Figure S6).

**Figure 4:**
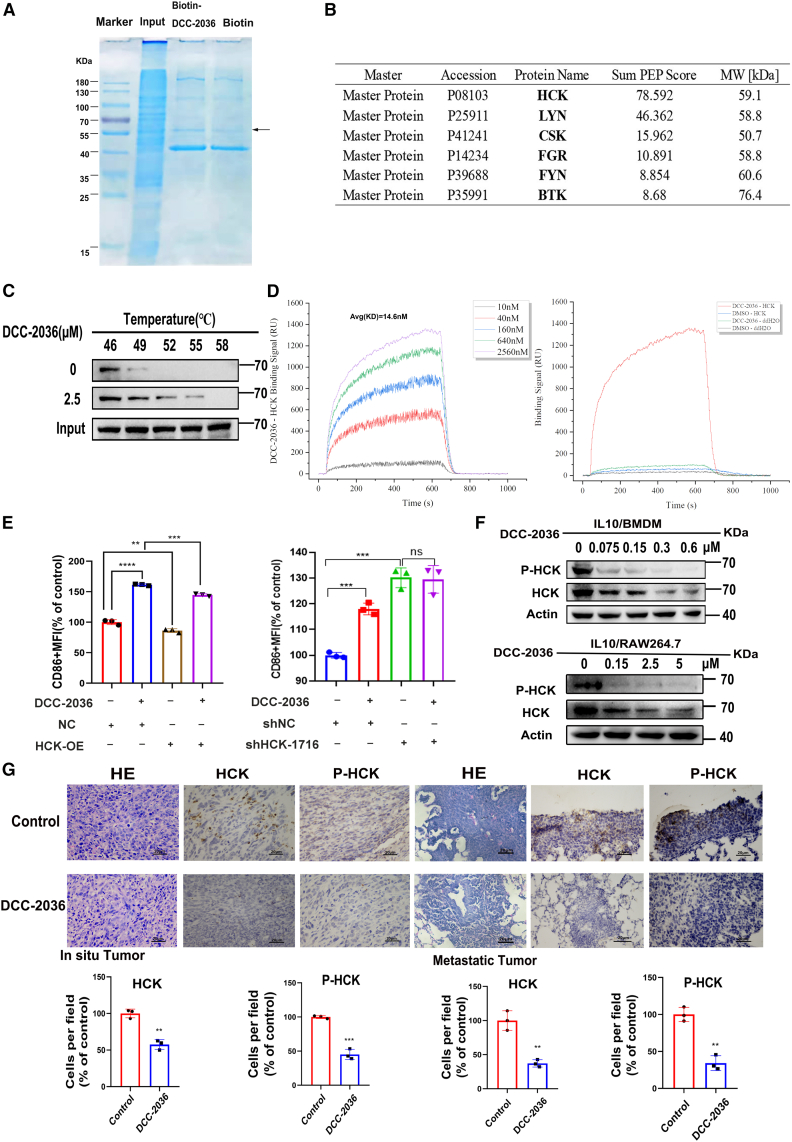
HCK is a key target of DCC-2036 that affects the polarization and function of tumor-associated macrophages (A) The biotin-labeled DCC-2036 was employed for the purification of proteins that exhibit binding affinity toward biotin-labeled DCC-2036, utilizing streptomycin affinity beads. Whole-cell lysate was served as a positive control (input), while biotin-purified proteins were utilized as a negative control (biotin). Subsequently, SDS-PAGE gel electrophoresis was conducted, followed by staining to visualize distinct bands. The arrow indicates the differentially expressed protein, and the presented result is representative of three independent replicates. (B) By extracting differential bands for mass spectrometry analysis, HCK, LYN, CSK, FGR, FYN, and BTK were initially identified as tyrosine kinases exhibiting high affinity toward DCC-2036. (C) Cellular thermal-shift assay (CETSA) of DCC-2036 with HCK. After treatment with 2.5 μM DCC-2036, the intracellular HCK protein stability in IL-10/RAW264.7 cells was enhanced. The input cell lysate is not subjected to thermal treatment. (D) Concentration-gradient-specific binding curves of the HCK protein were obtained at various concentrations using the immobilized phase DCC-2036. The interaction between HCK protein at a concentration of 2,560 nM and DCC-2036 was assessed on the chip. (E) To assess the level of HCK in M2-type RAW264.7 cells, we either overexpressed or silenced HCK using shRNA. Subsequently, the cells were treated with or without 2.5 μM DCC-2036, followed by flow cytometry analysis to evaluate CD86 expression as an indicator of M1 polarization. (F) The protein expression levels of HCK and phosphorylated HCK (p-HCK) were assessed in IL-10/BMDM cells and IL-10/RAW264.7 cells treated with varying concentrations of DCC-2036 using WB. (G) Immunohistochemical assessment of HCK and P-HCK expression in tumor tissues *in situ* and lung nodules. Scale bars, 20 μm.

Next, to further elucidate the key target of DCC-2036 on TAM polarization and function, we performed fishing experiments in TAMs to identify potential protein interactions with DCC-2036. In the IL10-induced M2-type BMDM model, significantly altered proteins with molecular weights ranging from 55 to 70 KDa were observed in the Coomassie brilliant blue-stained SDS-PAGE gel (Figure 4A). After conducting protein mass spectrometry analysis on the gel strips obtained from this location, it was observed that the biotin-DCC-2036 group exhibited differential tyrosine kinases, including HCK, LYN, CSK, FGR, FYN and BTK when compared to the biotin group. Most important of all, HCK displayed the highest score among them (Figure 4B). To investigate the direct interaction between DCC-2036 and HCK, we performed cellular thermal-shift assay (CETSA). Co-incubation of RAW264.7 cells with DCC-2036 resulted in a significant enhancement of intracellular HCK’s thermal stability (Figure 4C), confirming the direct binding between DCC-2036 and HCK within the cellular context. Additionally, surface plasmon resonance (SPR) experiment revealed a robust interaction between DCC-2036 and HCK protein, exhibiting an affinity constant of 14.6 nM (Figure 4D).

In order to further investigate the role of HCK as a crucial target in the action of DCC-2036 on TAMs, we treated M2-type RAW264.7 cells with either overexpressed or silenced HCK, followed by treatment with or without 2.5 μM DCC-2036. Subsequently, flow cytometry analysis was conducted on the collected cells. The findings revealed that, upon exposure to DCC-2036, there was an upregulation of the M1 polarization marker CD86. However, knockout of HCK hindered any further induction of CD86 upregulation by DCC-2036, whereas overexpression of HCK in M2-type RAW264.7 cells reversed the induction of CD86 upregulation (Figure 4E). Furthermore, WB experiments were conducted to assess the expression levels of p-HCK and total HCK in M2-type BMDM cells and RAW264.7 cells treated with varying concentrations of DCC-2036. The results demonstrated a concentration-dependent downregulation of both p-HCK and total HCK, with a more pronounced decrease observed in p-HCK (Figure 4F). Similarly, IHC analysis was employed to evaluate the expression levels of p-HCK and total HCK in tumor tissues from the *in situ* TNBC model as well as the lung metastasis nodes of TNBC. Compared to the control group, significant reductions were observed in both p-HCK and total HCK expression levels following treatment with DCC-2036 (Figure 4G). Consequently, we determined that DCC-2036 may potentially modulate the polarization changes of TAMs in TNBC by selectively targeting HCK.

### Knocking down HCK induces TAM repolarization and subsequently inhibits TNBC

To further explore the role of HCK in the immune regulation of TNBC, we first evaluated the effect of HCK knockdown in TAMs on the repolarization of TAMs. qPCR experiments demonstrated a reduction in mRNA expression of M2 markers CD163, VEGF, and ARG in IL-10/BMDM upon HCK knockdown. Conversely, the mRNA expression of M1 markers IL-23 and IL-6 increased (Figure S7A). Furthermore, through the implementation of immunofluorescence assay (Figures S7B and S7C) and WB experiment (Figure S7D), we observed a significant reduction in both average fluorescence intensity and protein expression levels of M2 markers in IL-10/BMDM upon HCK knockdown, accompanied by an increase in the expression levels of M1 markers. These findings provided evidence that downregulation of HCK facilitated the repolarization of IL-10/BMDM toward the M1 phenotype. Moreover, the co-cultured Transwell assay demonstrated that co-culturing with IL-10/BMDM treated with HCK knockdown as well as their CM production significantly reduced the migration and invasion activities of MDA-MB-231 and 4T1 cells compared to the NC group (Figure S7E). Repeated experiments were also conducted on IL-10-treated RAW264.7 cells (Figures S8A–S8E), yielding consistent findings. Ultimately, we concluded that downregulation of HCK could induce repolarization of TAMs toward the M1 phenotype, thereby suppressing migration and invasion of TNBC cells. To investigate the potential of TAM repolarization as a therapeutic strategy for inhibiting TNBC, we established an *in vivo* co-implanted model by co-injecting TAMs and 4T1 cells. *In vitro*, IL-10/RAW264.7 cells treated with shNC, shHCK-704, or shHCK-1716 were mixed with 4T1 cells and subcutaneously injected into the mammary fat pad of mice. The results demonstrated that tumor volume was significantly reduced in the co-implanted mouse model treated with shHCK-treated IL-10/RAW264.7 cells compared to the control group, with the most pronounced effect observed in mice treated with shHCK-1716-treated IL-10/RAW264.7 cells. Furthermore, there were no significant differences in body-weight changes among the three groups of mice (Figures S9A and S9B). The above results indicated that knocking down HCK on TAMs can induce their repolarization toward M1 phenotype, thereby inhibiting TNBC growth *in vivo*.

Considering the role of HCK in promoting tumor development and its limited expression on tumor cells,^29^ we hypothesize that silencing HCK specifically in TNBC cells may confer similar anticancer effects. Surprisingly, HCK knockdown in TNBC cell line 4T1 could not inhibit the cell viability, the colony-forming ability, or the migration and invasion capability of 4T1 cells (Figures S9C and S9E). Concurrently, 4T1 cells treated with shNC and shHCK were subcutaneously implanted into the fat pad of mice, and comprehensive tumor growth data were meticulously recorded. Intriguingly, our findings revealed no statistically significant disparities in terms of tumor growth rate, volume, or weight alteration between the NC group and the shHCK group (Figure S9F). These compelling results further substantiated that downregulation of HCK in TNBC cells failed to elicit an inhibitory effect on TNBC, indirectly indicating that the regulation of HCK on TNBC was mainly dependent on immune cells.

### Knockout of HCK inhibits the growth and metastasis of TNBC *in vivo* by promoting the repolarization of TAMs and enhancing anti-tumor CD8^+^ T cell immunity

**Figure 5:**
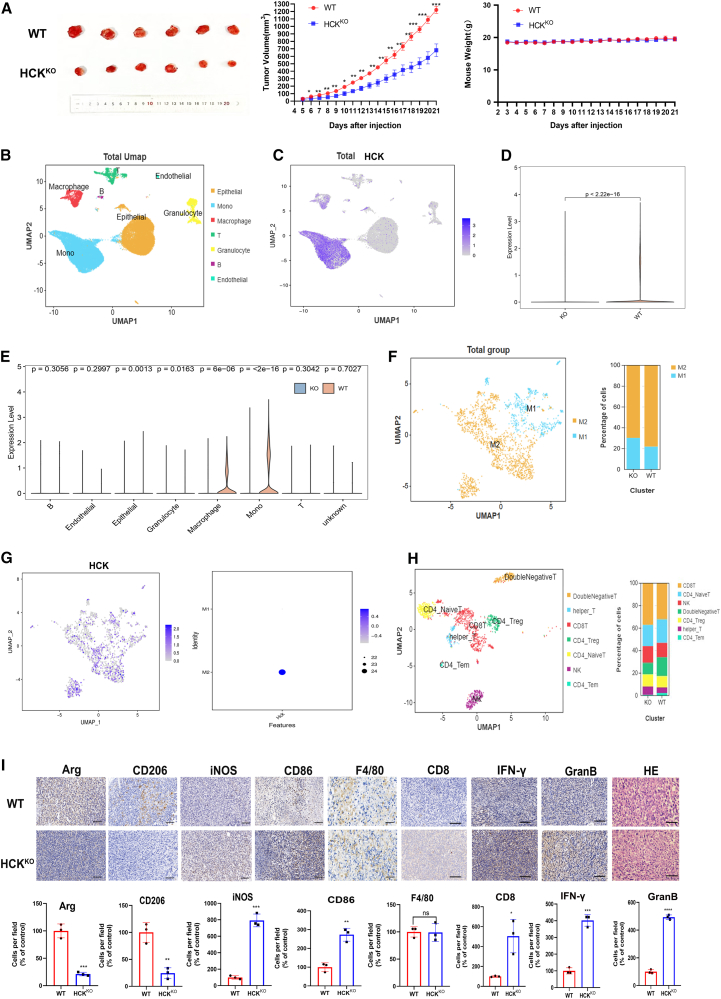
Knockout of HCK can induce repolarization of TAMs, thereby augmenting CD8^+^ T cell-mediated anti-tumor immunity (A) Photographs depicting tumor growth in wild-type (WT) mice (top) and HCK-knockout (HCK^KO^) group (bottom) were captured; 2 × 10^6^ 4T1 cells were implanted into the subcutaneous mammary fat pad of both WT and HCK^KO^ mice, and data regarding tumor progression were recorded. The WT group comprised *n* = 6 mice, while the HCK^KO^ group also consisted of *n* = 6 mice. Changes in tumor volume in mice; weight change curve in mice. (B) Two groups of tumor tissues, WT (*n* = 2) and HCK^KO^ (*n* = 3), were subjected to single-cell sequencing data analysis in order to investigate the cellular composition within tumor tissues. Subsequently, cell-clustering analysis was performed on the immune microenvironment. Cell subgroups were annotated based on specific markers, leading to the generation of an overall UMAP plot. (C) UMAP visualization of the distribution pattern of HCK gene expression. (D) Differences in HCK gene expression between WT and HCK^KO^ groups. (E) Differences in HCK gene expression in various cell types between WT and HCK^KO^. (F) UMAP plot depicting the comprehensive distribution of M1 and M2 macrophages in tumor tissue; bar graph illustrating the intergroup ratio of M1 and M2 macrophages. (G) Comprehensive UMAP plot illustrating the distribution of gene expression for HCK in macrophages; dot plot depicting the gene expression profile of HCK in macrophages. (H) UMAP plot depicting the comprehensive distribution of distinct T cell subgroups in tumor tissue; bar graph illustrating the relative proportions between different T cell subgroups across various groups (WT and HCK^KO^). (I) IHC experiments were conducted to assess the expression of macrophage and T cell markers in tumor tissues obtained from two distinct groups of mice, namely WT and HCK^KO^. Scale bars, 20 μm.

To validate the hypothesis that the *in vivo* ablation of HCK can curtail TNBC progression by orchestrating the repolarization of TAMs, we developed a CRISPR-Cas9 knockout HCK mouse model. Subcutaneous inoculation of 4T1 TNBC cells into the mammary fat pads was conducted in both wild-type (WT) and HCK-knockout (HCK^KO^) mice, with tumor size being monitored and documented. Following thorough observation, measurement, and subsequent data analysis, we observed a marked reduction in tumor volume in HCK^KO^ mice compared to their WT counterparts without a detectable alteration in body weight (Figure 5A). The above results indicated that the knockout of HCK could inhibit the growth of TNBC cells in mice. To delve into the intricate nexus between HCK and TNBC immune regulation, two sets of tumor tissues from mice were preserved for the single-cell sequencing analysis. The uniform manifold approximation and projection (UMAP) and gene-expression bubble map were plotted according to each cell-type marker (Figures 5B and S8A). It was found that HCK was mainly expressed in mononuclear macrophages and there was a significant downregulation in the HCK^KO^ group compared WT group (Figures 5C–5E). Moreover, M1 and M2 macrophages were characterized using UMAP plots and bubble map (Figures S10B and S10C). Analysis of the intergroup-ratio bar charts of M1 and M2 subtypes revealed a significant increase in the M1 subtype and a marked decrease in the M2 subtype in the HCK^KO^ group (Figure 5F). An examination of the overall HCK gene-expression UMAP plot and gene-expression dot plot in macrophages indicated that HCK was primarily expressed in M2 macrophages (Figure 5G). To elucidate the interplay between HCK silencing and T cell immune modulation, we meticulously constructed the UMAP plot and bar chart across diverse T cell subsets, utilizing specific markers for these populations. Our analysis revealed a significant augmentation in the proportion of CD8^+^ T cells in the HCK^KO^ cohort (Figures 5H and S10D).

In addition, immunohistochemical assays were conducted to elucidate the expression of macrophage polarization markers, as well as the counts and activation markers of CD8^+^ T cells, within tumor specimens from the two distinct mouse cohorts. The expression levels of M2 markers (ARG and CD206) were markedly attenuated in the tumor tissues of HCK^KO^ mice. The expression of M1 polarization markers, such as iNOS and CD86, was significantly upregulated in the HCK^KO^ group. However, no appreciable differences were discerned in the expression of the macrophage pan-marker F4/80 between the WT and HCK^KO^ groups. Furthermore, a significant increase in the expression of T cell markers, including CD8, IFN-γ, and granzyme B, was observed in the HCK^KO^ group (Figure 5I). Flow cytometric analysis and immunofluorescence studies further revealed similar results (Figures S11A–S11D). Collectively, these data suggested that the HCK^KO^ potentiated the CD8^+^ T cell response by redirecting TAMs toward an M1 phenotype, thereby exerting inhibitory effects on the growth of TNBC *in vivo*.

To explore whether the silencing of HCK could also impede TNBC metastasis by triggering the repolarization of TAMs *in vivo*, we established a mouse model of TNBC lung metastasis by injecting the 4T1-luc stable cell line into WT and HCK^KO^ mice via the tail vein. The experiment was conducted for 30 days. The IVIS *in vivo* imaging results revealed that the lung metastatic signals in the HCK^KO^ group were notably attenuated compared to those in the WT group (Figure S12A). Upon examination of the H&E-stained panoramic scan images of lung tissue sections from the three WT mice, a total of 296 lung nodules were counted, whereas, in the three HCK^KO^ mice, only 49 were observed. The HCK^KO^ group exhibited a significantly lower number of lung nodules than the WT group (Figure S12B), suggesting that the knockout of HCK could inhibit the lung metastasis of TNBC.

Based on the conclusion that knocking out HCK could enhance the anti-tumor CD8^+^ T cell immunity against TNBC, we speculated that knocking out HCK may increase the therapeutic efficacy of ICB against TNBC. The results confirmed that the use of programmed cell death ligand 1 (PD-L1) inhibitor atezolizumab alone and knockout of HCK exhibited anti-tumor effects to some extent. However, the combined therapy of HCK^KO^ with PD-L1 inhibitor was more potent in inhibiting tumor growth (Figures S12C and S12E). Additionally, there were no significant differences in weight fluctuations among the mice in various groups (Figure S12D), suggesting that knocking out HCK could enhance the response to ICB without adding burden to the mice.

### DCC-2036 potentiates the anti-tumor CD8^+^ T immune response in TNBC by modulating the production and secretion of IL-10 from TAMs

To elucidate the underlying mechanism by which DCC-2036 impedes the progression of TNBC through modulation of TAMs, we initiated an exploratory transcriptomic analysis in M2 BMDMs treated with/without DCC-2036. The volcano-plot analysis revealed a total of 4,035 genes displaying significant alterations in expression, with 1,855 genes upregulated (denoted by red dots) and 2,180 genes downregulated (denoted by green dots) following DCC-2036 administration. Gray dots represented genes with unchanged expression levels (Figure S13A). Notably, the clustering heatmap showed that treatment with DCC-2036 markedly enhanced the expression of eight typical M1 macrophage markers (CD86, tumor necrosis factor [TNF]-α, ANGPTL2, ITGAX, CCL3, IL-7R, IL-1α, and TREM1) while concurrently suppressing the expression of 19 common M2 macrophage markers (CCL17, MS4A4A, CCL22, CCR2, AIF1, MRC1, MAF, C3AR1, MSR1, LILRB4, FCGR2B, MMP9, CCL4, GRB2, ARG1, CSF1R, IL-10, CD163, and SIRPA) (Figure S13B). The result once again showed a pronounced shift of M2 macrophages toward an M1-like phenotype. Subsequently, Gene Ontology (GO) enrichment analysis revealed a significant correlation between the differentially expressed genes and the secretion of cytokines, such as IFN-β and IFN-γ, which are pivotal in T cell-mediated immune responses (Figure S13C). Furthermore, Kyoto Encyclopedia of Genes and Genomes (KEGG) enrichment analysis indicated enrichment in the chemokine signaling pathway and cytokine-cytokine receptor interaction signaling pathways (Figure S13D).

Following the revelation of a significant correlation between DCC-2036 and secreted factor-related pathways via transcriptomic analysis, we advanced our investigation by performing a secretomics examination on M2 BMDM cells treated with/without DCC-2036. The resulting volcano plot revealed a total of 2,747 upregulated and 2,406 downregulated secreted proteins, whereas proteins exhibiting minimal expression alterations were denoted by gray dots (Figure S13E). From the clustering diagram of secreted proteomics (Figure S13F), we also found three upregulated M1-type polarization indicators of changes in secreted proteins after DCC-2036 treatment, namely IRF5, NCF1, and RBPJ, and 12 downregulated M2-type polarization indicators of secreted proteomics, namely CCL4, CSF1R, LCN2, MMP9, TGFβ1, CCL2, LILRB4, GRB2, FN1, ITGAM, IL-10, and FCGR2. To elucidate the pivotal secreted factors that potentially modulate the function of TAMs (enhancing CD8^+^ T immunity), we constructed a Venn diagram (Figure S13G) incorporating markers indicative of macrophage polarization, which corroborated alterations within the BMDM transcriptome and secretome. CSF1R, IL-10, LILRB4, MMP9, and CCL4 between the transcriptomic and secretomic profiles had pronounced differences in expression levels. It has been documented that IL-10 secretion is not only associated with macrophage polarization but also with T cell immune suppression.^30^ Moreover, we found that the concentration of IL-10 in culture medium of DCC-2036-treated M2 macrophages was significantly lower than that in the control group by ELISA (Figure S13H). PCR analysis further revealed a marked decrease in the mRNA expression levels of IL-10 in M2-type RAW264.7 cells treated with DCC-2036/shHCK (Figures S13I and S13J). Consequently, it was inferred that DCC-2036 potentiated the anti-tumor CD8^+^ T immune response in TNBC by modulating the production and secretion of IL-10 from TAMs.

### DCC-2036 induces the repolarization of TAMs to M1 type through the regulation of metabolism reprogramming by the HCK-AKT/mTOR-GS-HIF1α axis

In recent years, it has been reported that the phenotypic transformation of TAMs is closely related to the metabolic reprogramming of TAMs. M1-type macrophages exhibit enhanced glycolysis and reduced oxidative phosphorylation compared to more oxidative M2-type macrophages, and modulating the polarization of TAMs by inducing their metabolic reprogramming to inhibit tumor progression is a promising therapeutic strategy.^31^^,^^32^ Targeting GS was found to reprogram IL-10-stimulated macrophages and TAMs *in vitro* into a desirable M1-like phenotype characterized by a decrease in intracellular glutamine, an increase in succinate production via the γ-aminobutyric acid (GABA) bypass, activation of HIF1α, and an increase in glycolysis after GS inhibition. In addition, GS-inhibited macrophages showed enhanced ability to induce T cell recruitment and activation.^18^^,^^33^ Thus, inhibition of GS in macrophages may serve as an immune-metabolic strategy to reduce tumor growth and metastasis.

**Figure 6:**
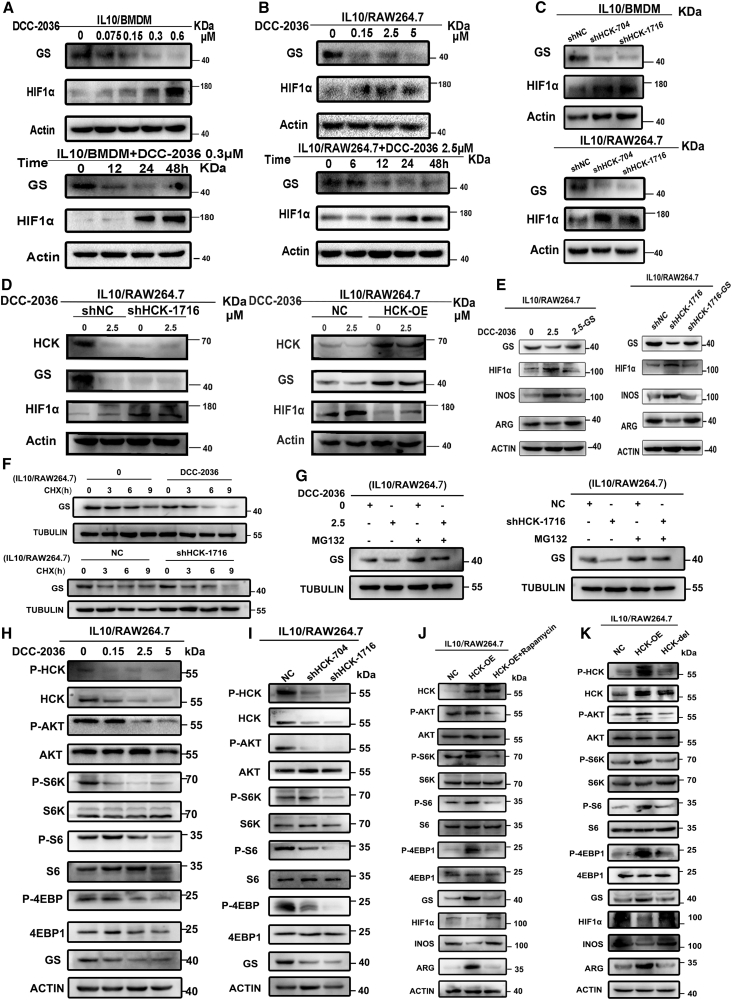
HCK-AKT/mTOR-GS-HIF1α axis-regulated metabolic reprogramming of TAMs mediates DCC-2036-induced repolarization of TAMs IL-10/BMDM cells (A) and IL-10/RAW264.7 cells (B) treated with DCC-2036 were collected for WB to analyze protein expression of GS and HIF-1α. The actin used in (A) and (B) was identical to Figures 1D and S2D because the collected proteins were the same. (C) Protein expression of GS and HIF-1α in IL-10/BMDM and IL-10/RAW264.7 cells treated with HCK knockdown. The actin used in (C) was identical to Figures S7D and S8D because the collected proteins were the same. (D) 2.5 μM DCC-2036 was applied to IL-10/RAW264.7 cells knocking down or overexpressing HCK for 48 h; cells were collected for WB to analyze HCK, GS, and HIF-1α protein expression. (E) IL-10/RAW264.7 cells were transfected with GS plasmid and then treated with 2.5 μM DCC-2036 or shHCK; cells were collected for WB to analyze the indicated protein expression. (F) IL-10/RAW264.7 cells were treated with DCC-2036 (2.5 μM) or shHCK. Cells were then treated with cycloheximide (CHX, 100 μg/mL) for 3, 6, or 9 h. Cells were collected for WB to analyze GS expression. (G) IL-10/RAW264.7 cells were treated with DCC-2036 (2.5 μM) or shHCK and then treated with MG132 (20 μM) for 8 h. Cells were collected for WB to analyze GS expression. IL-10/RAW264.7 cells treated with DCC-2036 (H) or shHCK (I) were collected for WB to analyze the protein expression of P-HCK, HCK, P-AKT, AKT, mTOR-associated markers, and GS. (J) 2 μM rapamycin was applied to IL-10/RAW264.7 cells overexpressing HCK for 48 h; cells were collected for WB to analyze the protein expression of HCK, P-AKT, AKT, mTORC-associated markers, GS, HIF-1α, and markers of macrophage polarization. (K) HCK plasmid, truncate missing the kinase domain of HCK, and vector plasmid were transfected into IL-10/RAW264.7 cells for 48 h, and then cells were collected for WB.

KEGG enrichment analysis of transcriptome sequencing in M2 BMDMs treated with/without DCC-2036 indicated enrichment in metabolic-related signaling pathways including the HIF-1 signaling pathway and biosynthesis of amino acids (Figure S11D). Thereby, to investigate whether DCC-2036 and HCK could cause metabolic reprogramming of TAMs by regulating GS and its downstream HIF1α and thus alter the phenotype of TAMs, we first performed WB experiments, which confirmed that DCC-2036 treatment decreased GS protein expression and increased HIF1α protein expression in M2 macrophages, and the effect was enhanced with the DCC-2036 concentration increased. In addition, DCC-2036 inhibited the expression level of GS and promoted the expression level of HIF1α in a temporal gradient (Figures 6A and 6B). Next, we similarly demonstrated by WB experiments that knockdown of HCK resulted in decreased GS protein expression and increased HIF1α protein expression in M2 macrophages (Figure 6C). More importantly, DCC-2036 could not further induce the downregulation of GS and upregulation of HIF1α after knocking down HCK, and the rescue experiments also suggested that the downregulation of GS and the upregulation of HIF1α by DCC-2036 could be reversed after HCK overexpression (Figure 6D). KEGG enrichment bubble plot of energy metabolism showed that the alanine, aspartate, and glutamate metabolism pathway was highly enriched after DCC-2036 treatment of M2 macrophages (Figure S14A). Meanwhile, the quantitative results of energy metabolism showed that, after DCC-2036 treatment of M2 macrophages, glutamine decreased; HIF1-α upstream metabolite such as succinic acid increased; key metabolites of glycolysis such as 3-phosphoglyceric acid, fructose-6-phosphate, glucose-6-phosphate, fructose 1,6-bisphosphate, phosphoenolpyruvate, pyruvate, and lactate increased; glucose level decreased; and oxidative phosphorylation-related metabolites such as adenine and ADP decreased. It was suggested that DCC-2036 promoted metabolic reprogramming of M2 macrophages from oxidative phosphorylation to glycolysis (Figure S14B). Therefore, we speculated that the HCK-GS-HIF1α axis-regulated metabolic reprogramming of TAMs might have mediated the DCC-2036-induced repolarization of TAMs to M1 type. Next, we further found that GS overexpression was able to rescue DCC-2036 and knockdown of HCK-induced downregulation of the M2 marker ARG, as well as upregulation of HIF1α and the M1 marker iNOS, by WB experiments (Figure 6E), and the co-immunoprecipitation (coIP) results showed that there was no interaction between HCK and GS (Figure S14C). It suggested that GS indirectly mediated the regulation of DCC-2036 and knockdown of HCK for downstream HIF1α. Then, we examined the expression level of GS in M2 macrophages by RT-qPCR and found that DCC-2036 and knockdown of HCK treatments did not alter GS mRNA expression levels (Figure S14D). Therefore, we wondered whether DCC-2036 and knockdown of HCK could regulate GS expression at the post-translational level. Post-translational regulation at the protein level is mainly regulated by the ubiquitination pathway. The results of the cycloheximide (CHX) assay showed that the half-life of GS proteins after DCC-2036 and knockdown of HCK was much shorter than that of the NC group (Figures 6F, S14E and S14F). Consistent with this, in M2 macrophages, treatment with ubiquitin-proteasome inhibitor (MG132) restored the reduction in GS protein induced by DCC-2036 and knockdown of HCK (Figure 6G). In addition, DCC-2036 and knockdown of HCK increased the ubiquitin level of GS (Figures S14J and S14K). The above results suggested that the downregulation of GS induced by DCC-2036 and knockdown of HCK depended on the ubiquitin-proteasome pathway.

We found that HCKs could regulate GS, but they could not interact with each other, which suggested that HCKs indirectly caused changes in GS. Then how did HCKs indirectly cause changes in GS? The differential genes regulated by HCK knockdown in M2 macrophages by KEGG enrichment analysis of transcriptomics were mainly associated with the PI3K-AKT signaling pathway (Figure S14G). It has been reported that mTORC1 could maintain GS stability by preventing ubiquitination and proteasomal degradation of GS.^34^ Therefore, we speculated whether HCK could mediate the GS stability through the PI3K-AKT-mTOR axis. To explore this, we confirmed by WB experiments that protein expression of P-HCK, HCK, P-AKT, mTOR-related markers, and GS was downregulated in M2 macrophages after treatment with DCC-2036 and knockdown of HCK (Figures 6H, 6I, S14H, and S14I), and the expression of these proteins was also upregulated after overexpression of HCK; this change was reversed when we further added the mTOR inhibitor rapamycin based on the overexpression of HCK (Figure 6J). To determine whether the kinase activity of HCK played a key role, we designed an HCK kinase-deficient truncated segment. We conducted WB experiments in M2 macrophages and found that protein expression of P-HCK, HCK, P-AKT, mTOR-related markers, GS, and HIF1α were reversed in the context of deficiency of the HCK kinase domain compared with the overexpression of HCK plasmid (Figure 6K). These results suggested that HCK mediated the GS stability through the PI3K-AKT-mTOR axis, depending on the kinase activity of HCK. In summary, HCK-AKT/mTOR-GS-HIF1α axis-regulated metabolic reprogramming of TAMs mediated DCC-2036-induced repolarization of TAMs.

### Clinical relevance of HCK and immune regulation in TNBC

**Figure 7:**
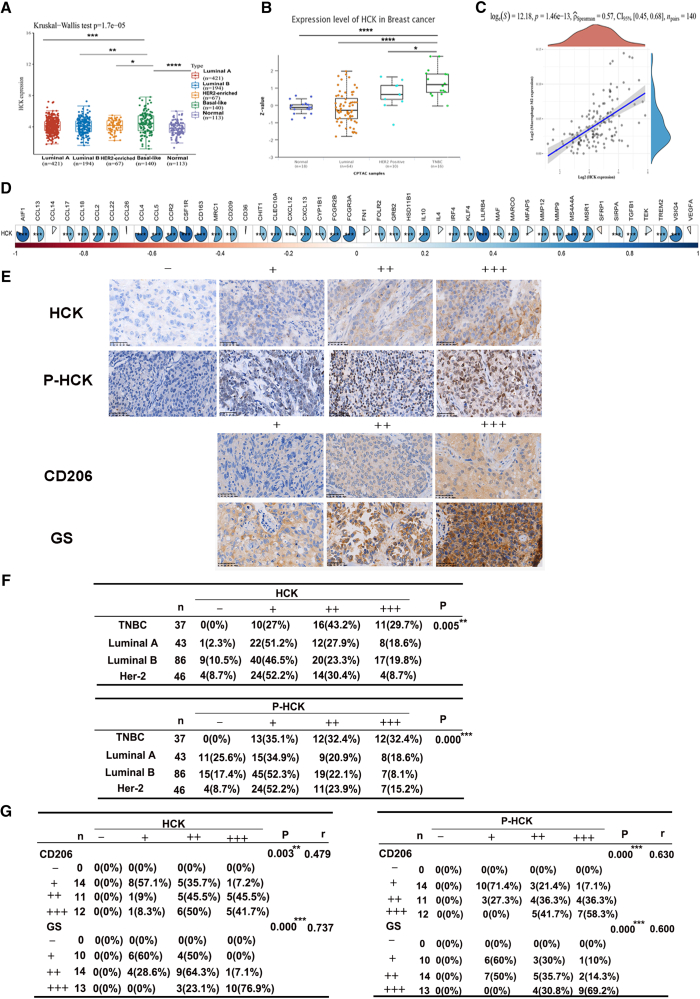
The immune regulation of HCK in TNBC exhibits a positive correlation with M2 macrophages and GS (A) By conducting a comprehensive analysis of the TCGA database, we meticulously examined the expression profiles of HCK in both normal tissues and diverse subtypes of breast cancer. (B) By mining the CPTAC sample database, we conducted an analysis on the expression levels of HCK in normal tissues as well as four distinct subtypes of breast cancer. (C) Using the Cibersort deconvolution tool, we predicted the immune cell infiltration in the BRCA dataset of the TCGA database and further investigated the correlation between HCK expression and immune cell infiltration in the basal-like subtype dataset. (D) By leveraging the BRCA dataset of the TCGA database, we investigated the association between HCK expression and M2 polarization markers in the basal-like subtype of breast cancer. (E) Representative immunohistochemical staining images of HCK, P-HCK, CD206, and GS were acquired from a cohort of 212 breast cancer patient specimens at the First Affiliated Hospital of University of South China. This cohort comprised 37 cases of TNBC subtype patients, 43 cases of luminal A subtype patients, 86 cases of luminal B subtype patients, and 46 cases of Her-2 subtype patients. Tissue sections were prepared by embedding the collected specimens followed by IHC analysis to assess the expression levels of HCK, P-HCK, CD206, and GS in each specimen. The expressions of HCK, P-HCK, CD206, and GS were categorized as negative (−), weakly positive (+), moderately positive (++), or strongly positive (+++). Scale bar, 200 μm. (F) The expression of HCK and P-HCK in TNBC, luminal A, luminal B, and Her-2 subtypes was assessed using the Kruskal-Wallis test. (G) The Spearman test method was employed to examine the correlation between HCK/P-HCK and CD206/GS in TNBC. ∗*p* < 0.05,∗∗*p* < 0.01,∗∗∗*p* < 0.001,∗∗∗∗*p* < 0.0001.

To explore the clinical relevance of HCK and immune regulation in TNBC, we first analyzed the HCK expression levels utilizing The Cancer Genome Atlas (TCGA) database and the CPTAC sample repository accessed through the ULCAN portal, revealing a substantial difference in HCK expression between normal and breast cancer tissues, with the HCK expression level being markedly elevated in the latter. Among these subtypes, the basal-like (predominantly encompassing TNBC) type presented the highest expression levels of HCK (Figures 7A and 7B). Using the deconvolution tool Cibersort to predict immune cell infiltration in the BRCA dataset from the TCGA database, it was found that the expression of HCK was significantly positively correlated with the number of M2 macrophages in the basal-like subtype dataset (Figure 7C). Through the extraction of the BRCA dataset from the TCGA repository, our analysis also revealed a significant positive correlation between the expression of HCK and the majority of M2 polarization markers in basal-like breast cancer (Figure 7D). These findings suggested that the immune-modulation function of HCK was closely associated with the polarization of M2 macrophages in TNBC.

Next, specimens of breast cancer were collected, totaling 212 cases, which included 37 cases of TNBC, 43 cases of luminal A, 86 cases of luminal B, and 46 cases of Her-2. These specimens were processed and examined for the expression of HCK, P-HCK, CD206, and GS by immunohistochemical methods (Figure 7E). The results revealed that there were significant differences in the expression of HCK and P-HCK among the TNBC, luminal A, luminal B, and Her-2 subtypes (*p* < 0.05), with HCK expression being significantly higher in TNBC compared to other subtypes (Figure 7F). Furthermore, statistical analysis indicated a significant positive association between the expression of CD206 and GS with that of HCK and P-HCK in TNBC (Figure 7G). All the results were consistent with our earlier study and indicated that HCK could be a potential target in TNBC immunotherapy.

### DCC-2036 enhances the therapeutic efficacy of ICB in TNBC

**Figure 8:**
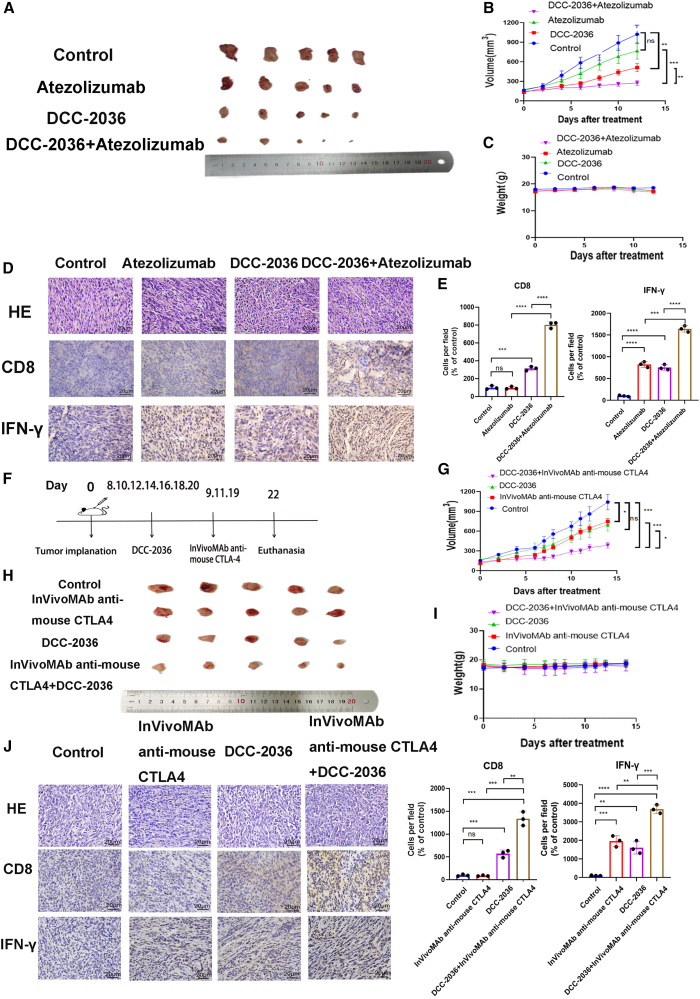
DCC-2036 synergistically enhances the therapeutic efficacy of ICB in TNBC (A) Representative tumor images of the control group, atezolizumab treatment group, DCC-2036 treatment group, and combination therapy of atezolizumab with DCC-2036 group. Balb/c mice were inoculated with 2 × 10^6^ 4T1 cells into the fourth mammary fat pad. Upon reaching a tumor volume of 100–200 mm^3^, the mice were randomly allocated into four groups: control group (*n* = 7), atezolizumab group (*n* = 6, administered at a dose of 5 mg/kg per week via intraperitoneal injection), DCC-2036 group (*n* = 7, administered at a dose of 50 mg/kg every other day via oral gavage), and atezolizumab + DCC-2036 group (*n* = 7). (B) Tumor growth kinetics in Balb/c mice were assessed by monitoring changes in tumor volume following administration of various treatments. (C) Weight-change profiles of Balb/c mice were monitored following the administration of various treatments. (D and E) Tumor tissues from different treatment groups were subjected to H&E staining, CD8 immunohistochemical staining, and IFN-γ immunohistochemical staining using a scale of 20 μm. (F) Representative tumor images were obtained from the control group, CTLA-4 inhibitor treatment group, DCC-2036 treatment group, and combination therapy of CTLA-4 inhibitor with DCC-2036 group. Balb/c mice were injected with 2 × 10^6^ 4T1 cells in the fourth mammary fat pad. Upon reaching a tumor volume of 100–200 mm^3^, the mice were randomly divided into four groups: control group (*n* = 5), CTLA-4 inhibitor group (*n* = 5; administered at a dose of 4mg/kg/mouse on days 9, 11, and 19 after tumor inoculation via intraperitoneal injection), DCC-2036 group (*n* = 5; administered at a dose of 40 mg/kg/mouse every other day by oral gavage), and CTLA-4 inhibitor + DCC-2036 group (*n* = 5). (G) The growth curve of tumors in mice. (H) The weight trajectory of mice. (I and J) Tumor tissues from different treatment groups were subjected to H&E staining, CD8 immunohistochemical staining, and IFN-γ immunohistochemical staining using a scale of 20 μm. The significance of the findings was assessed through t test analysis: ∗*p* < 0.05,∗∗*p* < 0.01,∗∗∗*p* < 0.001,∗∗∗∗*p* < 0.0001.

Currently, immunotherapy has emerged as a breakthrough in the treatment of cancer. Nonetheless, TNBC predominantly maintains a “cold” tumor profile, characterized by suboptimal response rates to ICB therapy. The heterogeneous mechanisms underpinning immune checkpoint inhibitor resistance are profoundly intricate, with modulation of the TME emerging as a pivotal element.^35^ Building upon our prior discoveries, we pursued the possibility that DCC-2036 may augment the efficacy of immune checkpoint inhibitors in the treatment of TNBC. Investigations revealed that the synergistic combination of a PD-L1 inhibitor atezolizumab with DCC-2036 demonstrated a more efficacious suppression of TNBC tumor growth compared to the monotherapy of either agent alone. This effect was achieved with a favorable toxicity profile and without precipitating significant weight fluctuations in experimental mice (Figures 8A–8C). Furthermore, immunohistochemical analysis of tumor tissues from treated mice across all groups highlighted an augmented presence of CD8^+^ T cells and a concomitant enhancement in their functional activity within TNBC tumor tissues following the combined therapy (Figures 8D and 8E). Similar results were obtained when DCC-2036 was combined with anti-CTLA-4 monoclonal antibody (Figures 8F–8J). In conclusion, DCC-2036 has the potential to augment the therapeutic efficacy of ICB therapy in TNBC.

## Discussion

The novel small-molecule TKI DCC-2036 (rebastinib), developed by Tufts Medical Center to target ABL1, is a third-generation TKI with a wider range of kinase inhibition currently undergoing clinical trials for potential cancer treatment.^22^^,^^23^ Our previous research indicated that DCC-2036 is a highly effective compound against TNBC. It effectively inhibits the proliferation, migration, and invasion of TNBC cells both *in vitro* and *in vivo* by targeting the tyrosine kinases AXL/MET and regulating downstream PI3K/Akt-NF-κB signaling pathways.^24^ Additionally, it suppresses tumor stem cells in TNBC by targeting the AXL-KLF5 positive-feedback loop, enhancing TNBC sensitivity to chemotherapy.^25^ While our previous studies focused on the direct inhibitory effects of DCC-2036 on tumor cells/stem cells, its impact on tumor immunity of TNBC and its relationship to TNBC suppression have not been explored. This study provides clear evidence that DCC-2036 can also induce anti-TNBC effects through immune-modulation functions.

Our research group has utilized flow cytometry, WB, and animal experiments to demonstrate that DCC-2036 has the ability to repolarize IL-10-induced M2 macrophages *in vitro* and TAMs in mouse tumor tissues *in vivo*, converting them into M1 type to elicit anti-TNBC effects. Additionally, DCC-2036 has been shown to enhance both the quantity and functionality of CD8^+^ T cells in TNBC. Notably, the anti-tumor efficacy of DCC-2036 is compromised in the absence of either macrophages or T cells, suggesting that DCC-2036 triggers T cell-mediated immunity to exert its anti-tumor properties, with macrophages playing a crucial role in activating the anti-tumor T cell immune response.

M1 macrophages have the ability to eliminate tumor cells through various mechanisms; however, tumor cells can effectively influence macrophages to transition toward the M2 phenotype using direct or indirect methods.^36^^,^^37^^,^^38^ Targeting M2 TAMs within the TME is considered a promising strategy for treating tumors.^39^ While current studies primarily focus on depleting M2 TAMs, such as blocking the CCL2/CCR2 recruitment pathway^40^ and interfering with other signaling pathways associated with M2 TAMs,^41^ our research introduces an alternative approach. This approach involves utilizing DCC-2036, a small-molecule compound that can easily target TAMs in the TME. DCC-2036 not only reprograms M2 macrophages to the M1 type without depleting them but also effectively harnesses macrophages to reconstruct an anti-tumor immune microenvironment. Animal experiments have demonstrated the safety of DCC-2036. Additionally, the use of immune checkpoint inhibitors has shown significant progress in enhancing anti-tumor immune responses. However, the efficacy of T cell checkpoint blockade therapy is limited in TNBC as a cold tumor.^42^^,^^43^ Our study reveals that DCC-2036 enhances immunotherapy by converting macrophages into an M1-like phenotype, thereby activating CD8^+^ T cells to suppress the growth and metastasis of TNBC. This synergistic effect enhances immune therapy when combined with the PD-L1 inhibitor atezolizumab or CTLA-4 monoclonal antibody, suggesting a promising combination therapy strategy for TNBC treatment. To date, the combination of the multi-kinase inhibitor DCC-2036 with the anti-PD-L1 monoclonal antibody atezolizumab has been systematically evaluated only in colorectal cancer (CRC). Our research group demonstrated that DCC-2036 markedly sensitizes CRC to atezolizumab.^44^ Compared with either monotherapy, the combination elicits a more robust and sustained anti-tumor response, as shown by significant reductions in tumor burden. Although this collaborative strategy has not yet been validated in the field of ER^+^ or HER2^+^ tumors, there is vast potential for future development. In conclusion, our research sheds light on the anti-tumor mechanism of DCC-2036 and positions it as a potential immunotherapy agent for TNBC.

An important finding of this study is that HCK is the key direct target of DCC-2036 in TAMs through target-capture experiments, SPR experiments, CETSA experiments, bioinformatics analysis, flow cytometry, etc. HCK a member of the SRC family of non-receptor tyrosine kinases (SFK), is predominantly expressed in myeloid cells and B lymphocytes, playing a crucial role in innate immune responses. Previous research has shown high expression of HCK in macrophages of colon cancer, linked to M2 polarization and poor prognosis.^45^ However, the specific relationship and regulatory mechanism between HCK and TNBC immune regulation remains unclear. Analysis of the TCGA database and clinical samples revealed elevated HCK levels in breast cancer, particularly in the TNBC subtype, suggesting HCK as a potential biomarker and therapeutic target for TNBC patients. Knockdown of HCK in TAMs *in vitro* and knockout of HCK *in vivo* mirrored the effects of DCC-2036 treatment, underscoring the importance of HCK in immune cells. Notably, knockdown of HCK in TNBC cells did not affect cell growth, invasion, or metastasis, reinforcing the immune cell-dependent role of HCK. Single-cell sequencing in TNBC tumor tissues of HCK^KO^ mice and WT mice showed high HCK expression in M2 macrophages, with HCK^KO^ primarily impacting macrophage repolarization and CD8^+^ T cell activation. Further investigation is warranted to explore whether DCC-2036 and HCK knockdown/knockout can also modulate other immune cells to inhibit TNBC.

Another important finding of this study is that we identified the HCK-AKT/mTOR-GS-HIF1α axis as a key regulator of TAMs’ metabolic reprogramming, leading to their repolarization into M1 type upon DCC-2036 treatment by transcriptome and metabolome analysis, knockout experiments, rescue experiments, etc. Recent literature has highlighted the close relationship between TAMs’ phenotypic transformation and their metabolic reprogramming.^31^^,^^32^ Metabolic immunotherapy emerges as a promising strategy to shift macrophage function toward an anti-tumor phenotype, offering hope in the battle against cancer. Transcriptome sequencing revealed enrichment of metabolic-related signaling pathways like biosynthesis of amino acids and HIF-1 signaling pathways, while metabolome analysis indicated changes such as decreased glutamine, increased succinate as upstream of HIF1α, elevated glycolytic metabolites, decreased glucose levels, and reduced metabolites linked to oxidative phosphorylation in M2 macrophages treated with DCC-2036, signifying their metabolic reprogramming from oxidative phosphorylation to glycolysis. GS is recognized as a metabolic checkpoint regulating glutamine production and inflammatory mediator release. In macrophages, IL-10-driven GS activity is associated with M2-like functions. Conditional GS deletion in macrophages promotes the generation of anti-tumor, M1-like TAMs by decreasing intracellular glutamine, increasing succinate via the GABA shunt, activating HIF1α, and enhancing glycolysis to inhibit metastasis.^18^^,^^33^ Our research revealed that HCK does not directly interact with GS. However, transcriptome analysis of HCK knockdown in M2 macrophages showed that the differential genes post HCK downregulation are primarily associated with the PI3K-AKT signaling pathway. Existing reports suggest that mTORC1 may preserve GS stability by inhibiting its ubiquitination and proteasomal degradation.^34^ Ultimately, we verified that HCK can regulate GS stability via the PI3K-AKT-mTOR signaling axis, contingent on the kinase activity of HCK. In addition, from the analysis of transcriptomic and secretomic we demonstrated that DCC-2036 potentiates the anti-tumor CD8^+^ T immune response in TNBC by decreasing the production and secretion of IL-10 from TAMs.

In the TME, metabolic reprogramming of TAMs fuels tumor growth and immune suppression, making TAM-targeted metabolic drugs crucial for cancer therapy. VISTA is highly expressed on TAMs. Blocking VISTA with antibodies reprograms TAMs from a pro-tumor M2 to a pro-inflammatory M1 phenotype.^46^ This occurs via the STAT pathway: anti-VISTA treatment enhances STAT1 activation (promoting M1) and suppresses STAT6 signaling (associated with M2).^47^ CLEVER-1, a myeloid checkpoint on TAMs, activates LXR/RXR and PPAR pathways via ligand binding, promoting lysosomal function and lipid clearance while inhibiting NF-κB and JAK-STAT signaling, thus driving M2 polarization.^48^ The peptide conjugate MACTIDE-V reprograms CD206^+^ TAMs by blocking YAP, enhancing phagocytosis, and altering gene expression, showing promise for TNBC immunotherapy.^49^ Our research found DCC-2036, an HCK inhibitor, reprograms TAM metabolism from glycolysis to oxidative phosphorylation via PI3K/AKT-mTOR-GS-HIF-1α regulation. To date, DCC-2036 (rebastinib) has been evaluated in clinical studies for both leukemia and solid tumors. A phase I clinical trial assessed the safety and preliminary efficacy of rebastinib in chronic myeloid leukemia (CML) patients harboring T315I mutations. Among 40 evaluable CML patients, eight attained complete hematological responses, including four with T315I mutations. These results demonstrate that rebastinib exhibits quantifiable biological activity even in the challenging T315I-mutated subgroup, providing early evidence of its molecular targeting capability.^50^Additionally, a phase Ib study established the recommended phase II dose (RP2D) for combination therapy: rebastinib at 50 or 100 mg twice daily, combined with paclitaxel or eribulin, showing favorable tolerability. Although the study enrolled HER2-negative metastatic breast cancer patients, the defined dose also has been extended to investigations in ovarian cancer and other solid tumors.^51^ Clinical trials of anti-VISTA and anti-CLEVER-1 antibodies are currently in early phase I/II stages, enrolling patients with advanced solid tumors. In contrast, MACTIDE-V remains in the preclinical research stage to date. Moving forward, combination regimens involving these TAM-reprogramming agents hold promise for enhancing therapeutic efficacy. Accelerated clinical translation of these candidates is warranted.

In addition, we are conducting research and development on derivative compounds of DCC-2036. Currently, only one compound has been discovered, CCT196969, which has a structural similarity of 78% to DCC-2036 and exhibits a slightly better therapeutic effect on TNBC than DCC-2036. Notably, its mechanism of action involves targeted inhibition of the HDAC5/RXRA/ASNS signaling axis in TNBC cells. Therefore, exploring derivative compounds of DCC-2036 may be a good future research direction.^52^

In summary, DCC-2036 targets the HCK-AKT/mTOR-GS-HIF1α axis in TNBC to modulate the metabolic reprogramming of TAMs, leading to the repolarization of TAMs toward the M1 phenotype, resulting in a decrease in IL-10 secretion, which enhances the immune response of anti-tumor CD8^+^ T cells and increases the sensitivity of TNBC to ICB therapy (Figure S15). The findings of this study may identify novel drug candidates and therapeutic targets for immunotherapy in TNBC.

## Materials and methods

For isolation and culture of BMDMs, isolation of bone marrow-derived monocytes from both lower limbs of female 6 to 8-week-old C57BL/6 mice was done according to the instructions of the bone marrow monocyte isolation kit. After 4 h of culture, we aspirated the nonadherent cells, and the fresh complete medium containing monocyte colony-stimulating factor (M-CSF) (#315-02, Peprotech, USA) was added to induce macrophage formation. 6 days later, to verify the purity of macrophages, we determined the expression levels of F4/80^+^ and CD11b^+^ via flow cytometry. Subsequently, the cells were harvested for the experiments.

For cell lines, the TNBC cell line 4T1 and murine macrophage cell line RAW264.7 were purchased from the Cell Bank at the Chinese Academy of Sciences (Shanghai, China). The 4T1 and RAW264.7 were inoculated in culture dishes at suitable densities and then cultured in Dulbecco’s modified Eagle’s medium (DMEM) supplemented with 10% heat-inactivated fetal bovine serum (FBS) (Biological Industries, Northern Kibbutz Beit Haemek, Israel) and 1% penicillin/streptomycin at 37°C with 5% CO_2_.

For macrophage polarization and stimulation, to obtain M2-polarized macrophages, RAW264.7 cells, and the BMDMs, they were cultured in the presence of 10 ng/mL IL-10 (#210-10010, Peprotech). With RT-qPCR, WB, and flow cytometry, expression levels of macrophage polarization markers were measured to identify whether polarization was successfully induced.

For RT-qPCR, RNA was extracted from the samples using TRIzol reagent (CoWin Biotech). The extracted RNA was reverse transcribed into cDNA (Thermo Fisher Scientific). RT-qPCR was performed using the following mouse primers (primer sequences are shown in Table S1) to test the mRNA levels of CD163, VEGF, ARG, IL-10, CCL5, iNOS, IL-23, and IL-6. The data were analyzed using the comparative CT method (ΔΔCT method).

For WB, cells were treated and subsequently harvested. Lysates were prepared using RIPA buffer supplemented with protease and phosphatase inhibitors (Beyotime Biotech). Proteins of different molecular weights were separated by SDS-PAGE and transferred onto polyvinylidene fluoride (PVDF) membranes (Millipore). 5% milk was used to block plates for 1 h. Primary antibodies were applied, followed by a washing step to remove unbound antibodies. The main primary antibodies used included CD206 (#20536-1-AP, Proteintech), ARG (#66129-1-Ig, Proteintech), iNOS (#18985-1-AP, Servicebio), HCK (#116001-AP, Proteintech), p-HCK (#ab61055, Abcam), GS (#ab176562, Abcam), HIF1α (#20960-1-AP, Proteintech), actin (#20536-1-AP, Proteintech), and then the horseradish peroxidase (HRP)-conjugated secondary antibodies (Servicebio) were incubated and the excess antibodies were washed off by PBST/PBS. Finally, the immunoreactive bands were tested using a chemiluminescence detection system.

Generation of stable cells used lentiviral infection. The 4T1 cells were stably transduced with luciferase lentivirus (Shanghai GeneChem). After a 24-h incubation, the medium containing the lentivirus was replaced with fresh complete medium, and the culture was continued for an additional 48 h. Subsequently, the cells were subjected to selection using 4 μg/mL puromycin, leading to the establishment of a stable 4T1-luciferase (4T1-Luc) cell line. Finally, we measured the transfection efficiency >80% with the chemiluminescence detector and the 4T1-Luc cells could be used for the following experiments.

Generation of RAW264.7 cells stably knocked down HCK using lentiviral infection. To test the infection efficiency of different short hairpin RNA (shRNA) (shHCK-1716 and shHCK-704), we verified the expression of HCK by WB and RT-qPCR, and we selected shHCK-1716 (Shanghai GeneChem) with high-efficiency inhibition of HCK expression. RAW264.7 cells were infected by the lentivirus-loaded shHCK-1716 RNA and no-load lentivirus (follow-up operations were the same those to establish 4T1-Luc). RAW264.7 cells stably knocked down of HCK were used in subsequent experiments.

For *in situ* tumor inductions and treatment experiments, to establish an *in situ* tumor model, 2 × 10^6^ 4T1 cells were injected into the fourth mammary fat pad of female Balb/c mice aged 6–8 weeks, either in their conventional or nude variants. The mice were numbered and randomly grouped. The tumors were allowed to grow until they reached a mean tumor volume of 80–100 mm^3^. Mice in the experimental group were gavaged 100 mg/kg DCC-2036 every other day, while mice in the control group were administered the same amount of vehicle (0.5% carboxymethylcellulose [CMC]/1% Tween 80) every other day. Tumor volume and mouse weight were measured. Tumor growth inhibition (TGI) was calculated following TGI (%) = [(Vcontrol − Vtreatment) × 100%]/Vcontrol.

In the combination-therapy experiments, mice in the DCC-2036 treatment group were gavaged with 50 mg/kg on alternate days. Atezolizumab (5 mg/kg) (#A2004, Selleck) was injected intraperitoneally on day 11, 15, and 19 post tumor cell injection. Additionally, anti-CTLA-4 monoclonal antibody (mAb) (#BE0164, BioXCell) treatments were administered intraperitoneally at a dosage of 4 mg/kg on days 9, 11, and 19 following tumor inoculation.

To establish the tumor cell and macrophage co-transplantation *in situ* tumor model, RAW 264.7 cells, polarized to an M2 phenotype, were exposed to 2.5 μM DCC-2036 for 48 h with untreated cells serving as controls. The cells were washed and collected, and then we co-injected 2 × 10^6^ 4T1cells and 1 × 10^6^ RAW 264.7 cells into 6- to 8-week-old Balb/c female mice. Mice were divided into two groups based on whether the macrophages were treated with DCC-2036 or not. Tumor volume and mouse weight were measured.

For the metastatic tumor inductions and treatment experiments, 6- to 8-week-old Balb/c female mice were inoculated with 2 × 10^6^ 4T1-luc cells via the tail vein and randomly grouped. Mice were gavaged 100 mg/kg DCC-2036 or vehicle every other day from the first day after tumor inoculation. Mouse weight was recorded until the experiment ended. Luciferase substrates (#HY-12591B, MCE) were prepared as indicated in the manual. Mice were anesthetized with 150–200 μL 4% chloral hydrate after intraperitoneally injection with luciferin (5 mg/kg) for 15–20 min. IVIS Spectrum (PerkinElmer) was used for live-animal imaging.

For the macrophage depletion experiments, the control group and the DCC-2036-treated (100 mg/kg) group were managed the same as previously described. The macrophage scavenger-treated and combination groups were injected with clodronate liposomes (Yeasen Biotechnology) administered at a volume of 150 μL, with a concentration of 5 mg/mL, on days 0 and 12.

The HCK^KO^ Balb/c mouse model (HCK−/− mice or HCK^KO^ mice) was constructed by Gempharmatech (Nanjing, China). The mice were mated upon request and we used PCR to identify the genotypes of mice. 6- to 8-week-old female HCK^KO^ mice and WT mice were randomly selected for *in vivo* experiments. All the animal experimentations were approved by the animal ethics committee of the University of South China (USC202211XS95, USC202211XS96).

For isolation of tumor-infiltrating cells, tumor tissues were dissected and finely minced using scissors on ice. The tissue clumps were filtered out with a 70-μm cell sieve. After filtering, the tissues were homogenized and digested using collagenase and hyaluronidase in a gentle MACS C tube. The cell suspension was harvested and washed with PBS.

For flow cytometry staining and analysis, after treatment, T cells and macrophage-associated markers were analyzed by flow cytometry. The cells were stained with a corresponding mixture of fluorescently labeled antibodies: CD4 (#553051, BD Biosciences), CD8 (#2425227, Invitrogen), CD69 (#2376162, Invitrogen), CD45 (#550994, BD Biosciences, #103114, Biolegend), CD3 (#557369, BD Pharmingen), CD11b (#557396, BD Pharmingen), F4/80 (#557396, BD Pharmingen), CD86 (#105028, Biolegend), and CD206 (#25-2061-82, Invitrogen). The stained cells were then measured using a BD FACSAria II flow cytometer, and the resulting data were processed with FlowJo software.

For ELISA, RAW264.7 cells, polarized to the M2 phenotype, were subjected to either DCC-2036 treatment or HCK knockdown and cultured for 48 h. Cell-culture medium was collected. Samples were diluted 100-fold using sample diluent solution, and IL-10 concentration was measured in accordance with the protocol of the ELISA kit (Boster, EK0417).

For IHC staining, paraffin sections were prepared from tumor tissues or lung tissues that were excised from mice. IHC of tumor sections was performed with an IHC kit according to the manufacturer’s instruction (#G1212, #G1206, Servicebio). ARG (#66129-1-Ig, Proteintech), CD206 (#AF301039, AiFang biological), iNOS (#GB11119, Servicebio), CD86 (#AF300280, AiFang biological), F4/80 (#GB11027, Servicebio), CD8 (#AF20211, AiFang biological), IFN-γ (#AF301097, AiFang biological), GranB (#AF301221, AiFang biological), HCK (#116001-AP, Proteintech), P-HCK (#ab61055, Abcam), and GS (#ab228590, Abcam) expression levels in tumor were examined.

H&E staining was conducted according to standard protocols. The slides were subsequently scanned with a Tissue FAXS Spectra slide scanner, and the number of lung nodules was quantified.

For immunofluorescence staining, the tumor tissues were made into frozen sections after OTC embedding. Sections were fixed in 4% paraformaldehyde at room temperature, and the subsequent operations were conducted similarly to the IHC. The frozen sections were stained with DAPI (Absin) for 10 min and protected from light. Finally, we sealed and took photos under a fluorescence microscope (Leica, Wetzlar, Germany). Antibodies used were F4/80 (#GB11027, Servicebio), CD206 (#60143-1-Ig, Proteintech), CD86 (#NBP2-25208, Novusbio), CD8 (#AF20211, AiFang biological), and CD69 (#sc373799, SantaCruz).

For fishing experiments, we harvested the M2-polarized BMDM cells and extracted cellular proteins. The extracted proteins were labeled with either biotin or biotin-DCC-2036, following the manufacturer’s instructions for streptavidin conjugation (Invitrogen). Proteins were electrophoresed on SDS-PAGE and stained with colloidal Coomassie brilliant blue (Mei5 Biotech). The differential bands were cut and prepared for mass spectrometry analysis.

For RNA sequencing and data analysis, cells were collected and dissolved in TRIzol (CoWin Biotech) according to the ratio recommended by the instructions. Sequencings were performed by RIBOBIO (Guangzhou, China) and Metware (Wuhan, China). Genes whose expressions were at significantly different levels between groups with |log2 (fold change) | > 1 and *p* < 0.05 were defined as differential-expression genes.

For proteomic analyses of secreted proteins, the secreted proteins from DCC-2036-treated and control M2-polarized BMDMs were collected. After processing according to the standard procedure, the database files obtained by MaxQuant were analyzed using Perseus software. After statistical analysis, proteins with significantly different enrichment levels at *p* < 0.05 and fold differences ≥2 were selected.

For cell energy metabolism analysis, the BMDM pretreatment was the same as for RNA sequencing. Analysis of cellular energy metabolism was performed in Metware (Wuhan, China). Fold-change values ≥1.5 or ≤0.67 or VIP (Variable lmportance in Projection) > 1 were identified differential metabolites between the groups.

For Transwell assay after co-culture, BMDMs and RAW264.7 were treated with IL-10 for 6 h and then cultured with DMEM without FBS for 48 h. Finally, the supernatant was collected as the CM. 5 × 10^4^ MDA-MB-231 cells or 4T1 cells were seeded on the upper chambers of the Transwell plates. 250 μL of CM and 250 μL of complete medium were added to lower chambers of Transwell plates. 2 days later, cell metastasis was observed. The cell invasion can be detected when Matrigel is added to the upper chamber of a Transwell plate. The experimental procedures of TNBC cells co-cultured with macrophages were similar to those described above. We added 500 μL of complete medium to the lower chambers of Transwell plates, and the mixture of 5 × 10^4^ TNBC cells and 5 × 10^4^ M2-type macrophages was seeded to the upper chambers of Transwell plates in this experiment.

For the clone-formation assay, 4T1 cells were infected with shRNA (shNC, shHCK-704, and shHCK-1716). 10^3^ 4T1 cells/well were seeded in six-well plates. After 7 days, they were dyed with 0.5% crystal violet and then photographed.

For the Cell Counting Kit 8 (CCK8), every well was seeded with 1 × 10^3^ 4T1 cells and cultured in an incubator overnight. The cells were divided into three groups: the shNC group, the shHCK-704 group, and the shHCK-1716 group. Six replicate wells were set up for each group. Cells were cultured for 0, 24, 48, 72, and 96 h. The CCK8 assay kit (#BMU106-CN, Abbkine) was employed to test cell viability following the manufacturer’s protocol.

Surface plasmon resonance (SPR) was employed to quantify the binding affinity between DCC-2036 and HCK. A photo-cross-linkable sensor chip (Betterways, China) was functionalized with 10 μM DCC-2036 (in DMSO) by microarray printing (BioDotTM-1520): 280-μm pitch, 180-μm-diameter spots, four replicates, 2.5 nL per spot, and five passes (total 12.5 nL). Covalent immobilization was achieved via UV-induced carbene insertion into C–H bonds. HCK (analyte) was prepared in PBS at five concentrations: 10, 40, 160, 640, and 2,560 nM.

For CETSA, the M2 polarized RAW264.7 was treated with DCC-2036 for 4 h. The cells were collected and the cell suspension was divided into five groups (each group includes 80 μL). The cell suspensions were heated for 3 min and the temperature gradient was from 46°C to 58°C. The cells were disrupted by repeated freeze-thawing in liquid nitrogen after the samples were cooled down to 25°C for 3 min. Protein was collected for WB analysis.

For CHX tracing experiments, M2 polarized RAW264.7 was treated with DCC-2036 or knockdown of HCK. We added 100 μg/mL CHX to the cells and the cell lysates were collected after 0, 3, 6, or 9 h of treatment. Finally, the expression of GS (#ab176562, Abcam) was measured by WB.

For proteasome inhibitor MG132 treatment experiments, the pretreatments of RAW264.7 cells were the same as described above. Cells were treated with MG132 (20 μM) for 4 h before the proteins were harvested. The expression of GS (#ab176562, Abcam) was tested by WB.

For coIP, cells were lysed with IP lysis on ice for 30 min to obtain the cell proteins. The 30-50 μL of cell lysate was used as the input group, and the remaining lysate was equally separated into two groups. The IP antibody was incubated with cell lysate overnight at 4°C. Streptavidin magnetic beads were washed with PBS and resuspended in IP lysis buffer. Then, 40 μL of streptavidin magnetic beads (Invitrogen) was added to each tube, and the mixture was incubated for 4 h at 4°C. The magnetic beads were deposited and washed with IP wash buffer another four times. 40 μL of SDS-PAGE loading buffer was added, mixed well, and then heated at 95°C for 5 min. The supernatant was collected for WB experiments.

For the carboxy-fluorescein-diacetate-succinimidyl-ester (CSFE)-labeling assay, 24-well plates were wetted with sterile PBS. The treatment group had 5 μg/mL anti-mouse CD3 antibody (#553057, BD Pharmingen) added. Antibody-coated plates were incubated overnight at 4°C. The mouse spleen was removed under sterile conditions, and the CD8^+^ T lymphocytes were isolated using microbeads (Miltenyi, 130-117-044) and LS columns (Miltenyi, 130-042-401) on the following day. 4 μL of prepared CFSE (#565082, BD Pharmingen) stock solution was added to the cell suspension, and the mixtures were incubated in the water bath at 37°C for 15 min. After incubation, cells were washed with PBS, and then the CD8^+^ T lymphocytes were cultured in expansion medium including 1 μg/mL anti-mouse CD28 (#553294, BD Pharmingen). Three days later, the cells were harvested to perform flow cytometry analysis.

For IHC analysis of human breast cancer samples, human breast cancer tissue microarrays were prepared from 212 paraffin-embedded tissues from the pathology department of the First Affiliated Hospital of University of South China. IHC was performed as described above. This study was approved by the institutional ethics committees of the First Affiliated Hospital of University of South China.

For bioinformatics analyses, data of basal-like-subtype breast cancer from the TCGA breast cancer dataset (BRCA) (https://www.cancer.gov/ccg/research/genome-sequencing/tcga) and the UALCAN database (https://ualcan.path.uab.edu/index.html) were analyzed. Single-cell RNA sequencing was performed by Gene De novo (Guangzhou, China).

For ADMET analysis of compounds, DCC-2036 was subjected to *in silico* pharmacokinetic, ADMET, and enzyme-mediated metabolism analyses. Molecular structures were prepared in ChemDraw and converted to SMILES and 3D formats for input into QikProp, ADMETlab 3.0, and NERDD/GLORYx platforms. ADMET profiling encompassed physicochemical properties, absorption, distribution, metabolism, excretion, and toxicity predictions, integrating multi-task machine-learning models and pathway-based mechanistic analyses (Tox21). CYP-mediated metabolism was predicted using CYPstrate for major isoforms (CYP1A2, 2A6, 2B6, 2C8, 2C9, 2C19, 2D6, 2E1, and 3A4), while SULT-mediated conjugation potential was evaluated via FAME 3 and GLORYx. Endpoints included substrate likelihood, probable reaction types (oxidation, hydroxylation, acetylation, demethylation, hydrolysis, and glucuronidation), and associated priority scores, which were systematically aggregated to infer metabolic stability, bioavailability, and drug likeness.

## Data and code availability

• The raw transcriptomics data generated in this study were deposited in the Sequence Read Archive under accession numbers PRJNA1139664 (https://www.ncbi.nlm.nih.gov/sra) and PRJNA1140543 (https://www.ncbi.nlm.nih.gov/sra).

## Acknowledgments

This work was supported by projects of the 10.13039/501100001809National Natural Science Foundation of China (81972487 and 82271506), the 10.13039/501100004735Natural Science Foundation of Hunan Province (2025JJ81042, 2024JJ9408, 2021JJ20039, 2022JJ70038, and 2023JJ60053), the 10.13039/100017695Health Commission of Hunan Province (20253572 and 202104070680), and the Hunan Provincial Clinical Medical Research Center for Drug Evaluation of major chronic diseases (2023SK4040).

## Author contributions

X.Z. and Y.S. initiated the idea and project. M.X., P.L., Z.C., and Jun Liu designed the experiments. R.H., J.C., Jun Li, and L.Y. advised on experimental designs and data analysis. J.Z., Xisha Chen, J.F., J.H., and Xiguang Chen analyzed the data. Y.L., Q.Z., and M.X. performed related experiments. Y.S, Y.L., Q.Z., and M.X. wrote and revised the manuscript. All authors read and approved the final manuscript.

## Declaration of interests

The authors declare no competing interests.

## Supplemental information

Document S1. Figures S1–S15 and Tables S1–S6

Document S2. Article plus supplemental information
