# Supplementary material for: DCC-2036 induces repolarization of TAMs to M1 type and enhances CD8+ T cell immunity in TNBC
Source: Mol Ther. 2025 Oct 24;34(2):985–1008. doi: 10.1016/j.ymthe.2025.10.042 (PMC12882329; doi:10.1016/j.ymthe.2025.10.042)
Supplement: Document S1. Figures S1–S15 and Tables S1–S6 [file mmc1.pdf]

YMTHE, Volume *34*

Supplemental Information

DCC-2036 induces repolarization of TAMs to M1 type and enhances CD8$^+$ T cell immunity in TNBC

Yuxin Liang, Qiting Zeng, Maoyu Xiao, Pei Li, Rongfang He, Zhangjie Chen, Jun Liu, Jingsong Cao, Jun Li, Liyang Yin, Jing Zhong, Xisha Chen, Jianbo Feng, Jun He, Xiguang Chen, Xuyu Zu, and Yingying Shen

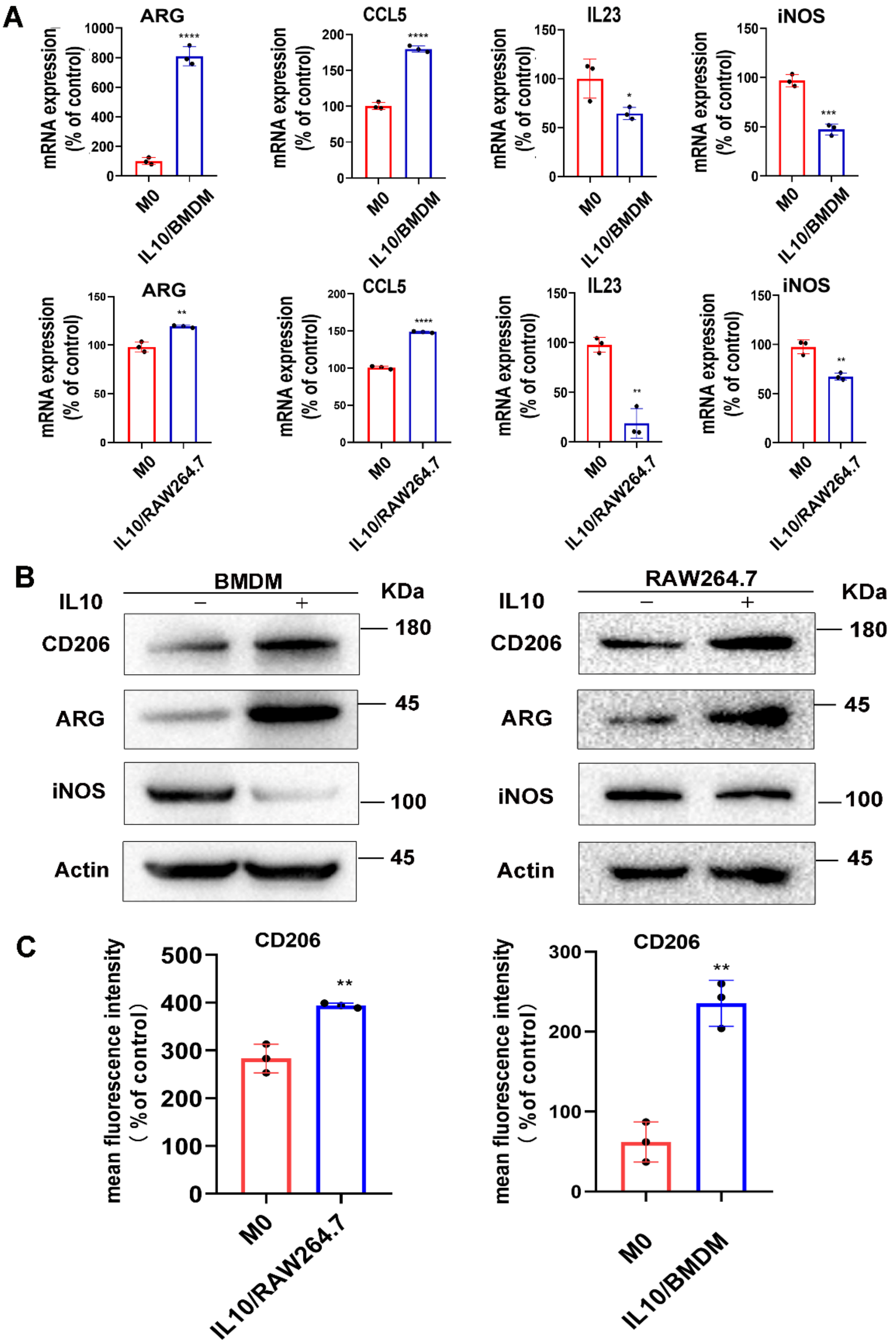

Figure S1 The effect of IL10 on macrophage polarization. We conducted experiments using macrophages (BMDM and RAW264.7) and macrophages that had been induced with IL-10 for 48 hours (IL-10/BMDM and IL-10/RAW264.7). (A) The mRNA expression of M1 and M2 polarization markers in BMDM and IL-10/BMDM was assessed using q PCR. (B) The mRNA expression of M1 and M2 polarization markers in RAW264.7 and IL10/RAW264.7 was assessed using q PCR. (C) The protein

expression of M1 and M2 polarization markers was assessed in IL-10/BMDM and IL-10/RAW264.7 cells using Western Blotting. (D) Flow cytometry analysis was performed to compare the expression of CD206 on BMDM, IL10/BMDM, RAW264.7 and IL10/RAW264.7 cells for subsequent statistical analysis, the results are shown in bar charts (n = 3, independent experiments). The statistical significance was determined by Student's t-test. *p<0.05， **p<0.01， ***p<0.001， ****p<0.0001.

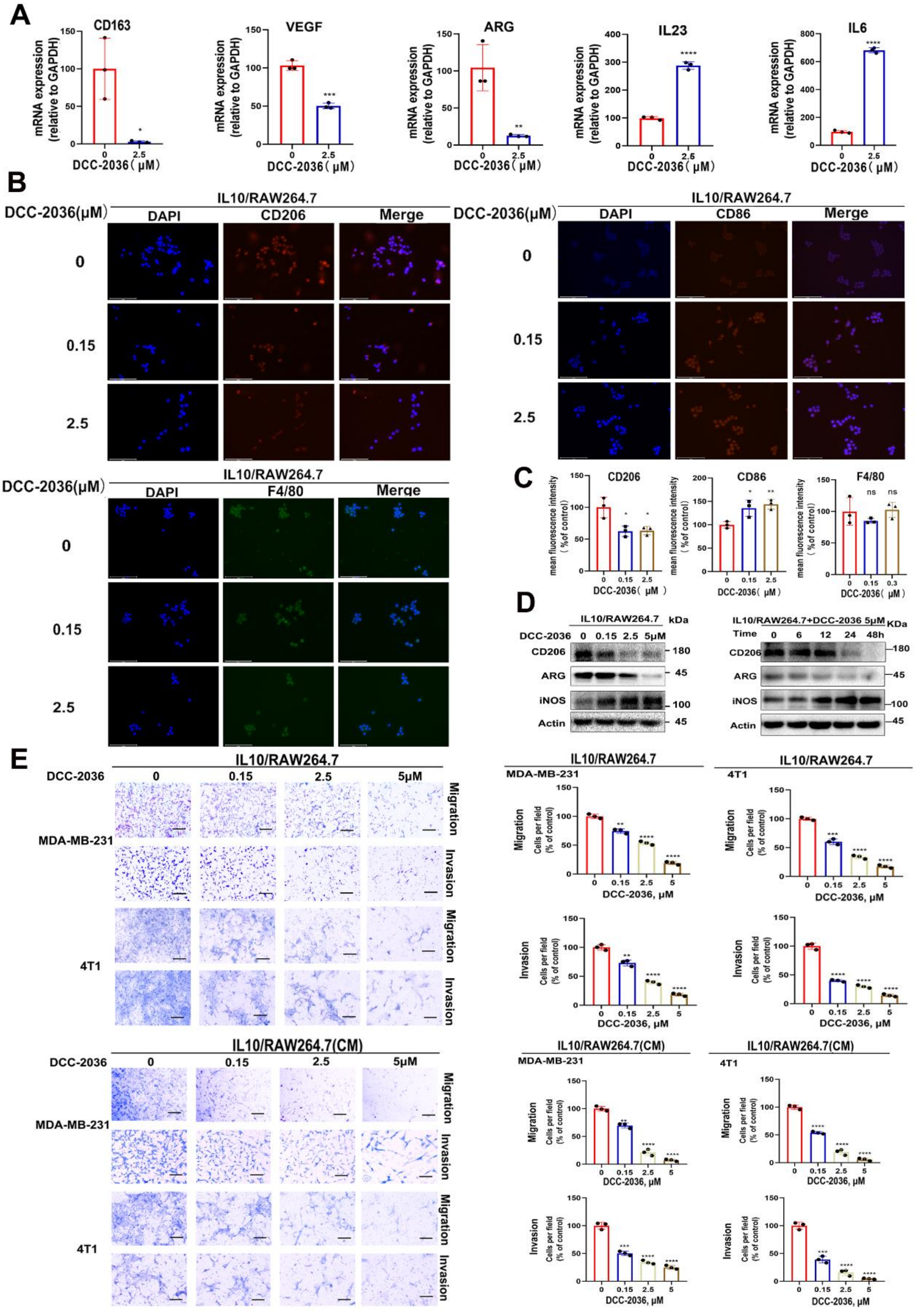

Figure S2. Effect of DCC-2036 on the polarization of M2-type macrophages (IL10/RAW264.7) and its effect on the migration and invasion of triple-negative breast cancer cells. IL10/RAW264.7 were treated with DCC-2036 with indicated concentration for 48 h. (A) Expression of M1 and M2 macrophage markers in M2-type macrophages treated with DCC-2036 (at a concentration of 2.5 μM) *in vitro* was detected by q PCR. (B) Immunofluorescence detection of mean fluorescence intensity

of M1 and M2 markers in IL10/RAW264.7 treated with different concentrations of DCC-2036. Scale bars, 75 μm (C) Former results are shown in bar charts (n = 3, independent experiments). The statistical significance was determined by Student's t-test. *p<0.05, **p<0.01, ***p<0.001, ****p<0.0001. (D) Different concentrations of DCC-2036 were added in IL10/RAW264.7 cells for 48 hours; DCC-2036 at a concentration of 2.5 μM was applied to IL10/RAW264.7 in a time gradient, Cells were collected for Western Blotting to analyze protein expression of M1 and M2 macrophage markers. (E) IL10/RAW264.7 treated with different concentrations of DCC-2036 were co-cultured with MDA-MB-231 and 4T1 cells; Conditioned medium (CM) of IL10/RAW264.7 cells treated with different concentrations of DCC-2036 was co-cultured with MDA-MB-231 and 4T1 cells. Scale bars, 50μm.The statistical significance was determined by Student's t-test, *p<0.05, **p<0.01, ***p<0.001, ****p<0.0001.

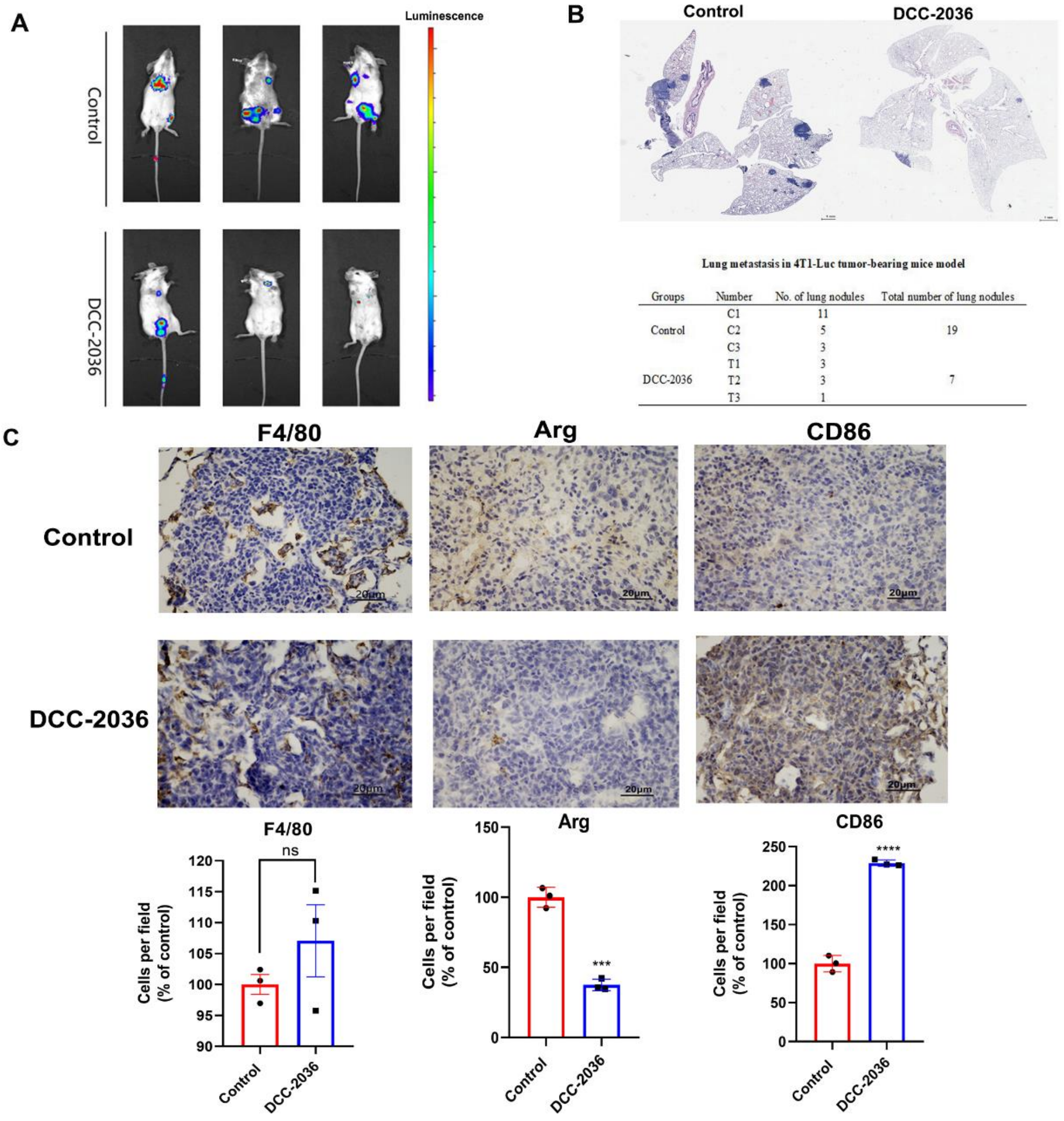

Figure S3. DCC-2036 inhibits triple-negative breast cancer metastasis *in vivo* by inducing polarization of tumor-associated macrophages. (A) A live imaging of tumor metastasis in Balb/c mice with control group (top) and treatment group (bottom), where n=3 for the control group and n=3 for the treatment group. The treatment duration is 14 days. (B) Representative images of mouse lung tissue stained with HE were obtained using panoramic scanning in both the control group (top, left) and treatment group (top, right). Table presenting the tumor nodule counts in mouse lung tissues for both the control group and treatment group. (C) Immunohistochemical assessment was performed to evaluate the expression levels of F4/80, ARG, CD86 within tumor nodules in lung tissue. Scale bars, 20 μm. The statistical results were visually represented using a bar chart, displaying the Mean ± SD with n = 3. The significance of the findings was assessed through t-test analysis, *p<0.05， **p<0.01， ***p<0.001， ****p<0.0001.

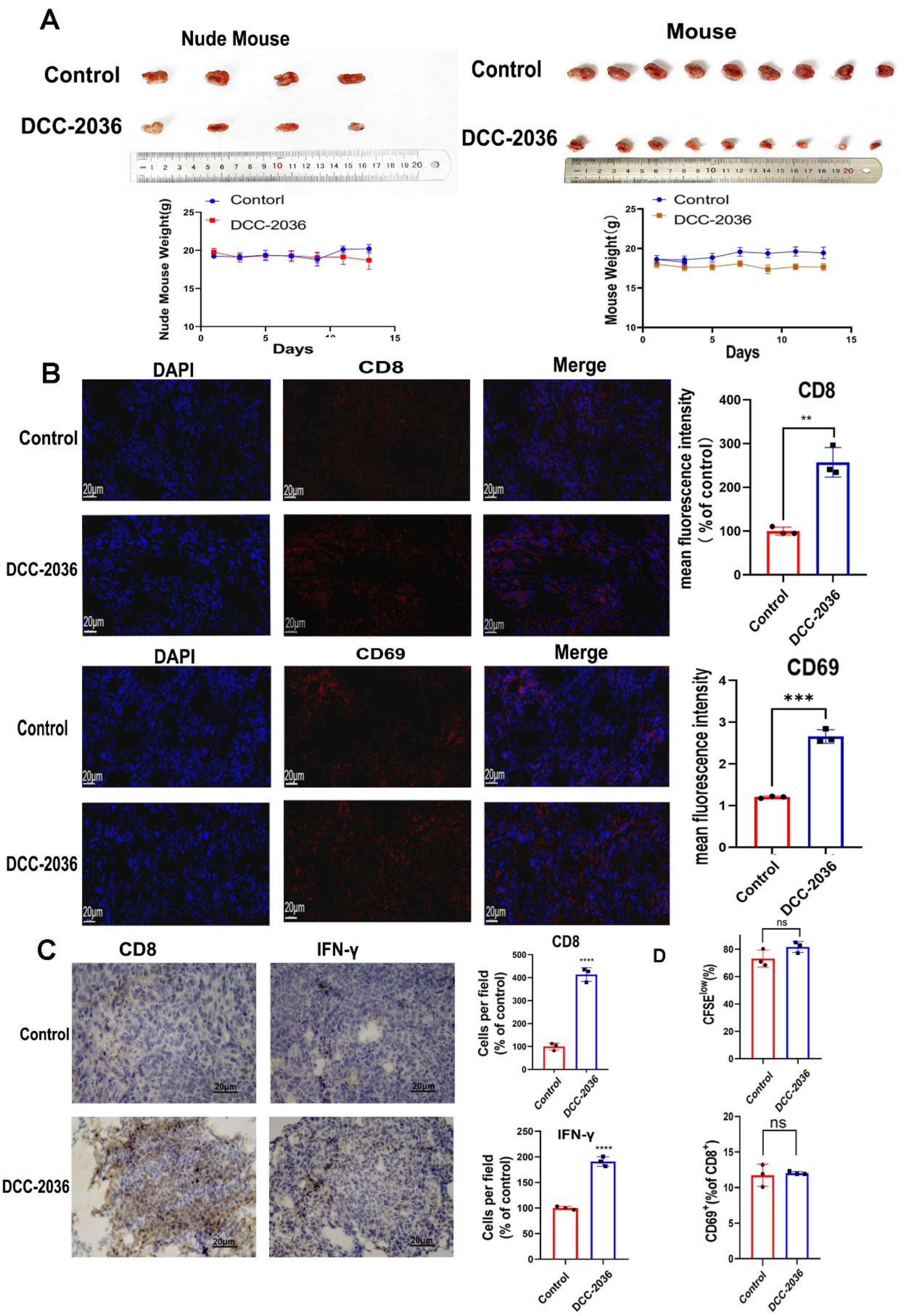

Figure S4. Supplementary data for Figure 3. (A) Images of collected tumor xenografts from Balb/c nude mice and Balb/c mice at the termination of the experiment; Weight change profiles of Balb/c mice and nude mice based on the measured data. (B) Subcutaneous transplantation of Balb/c mouse tumors was employed for immunofluorescence experiments to compare the expression levels of CD8 and CD69 in tumor tissues between the control group and mice subjected to DCC-2036 treatment.

(C) Immunohistochemistry (IHC) experiments were conducted on lung tissues obtained from Balb/c mice with metastatic tumor models (Figure S3) to compare the expression levels of CD8 and IFN-γ in the tumor tissues of two experimental groups. (D) The comparison of $CD8^+$ T cell proliferation (CFSE dilution assay) and $CD8^+$ T cell activity ($CD69^+$) following *in vitro* treatment with the control group (0 μM) and DCC-2036 (2.5 μM) was analyzed using flow cytometry (n=3, t-test, mean ± SD; ns, no statistical significance).

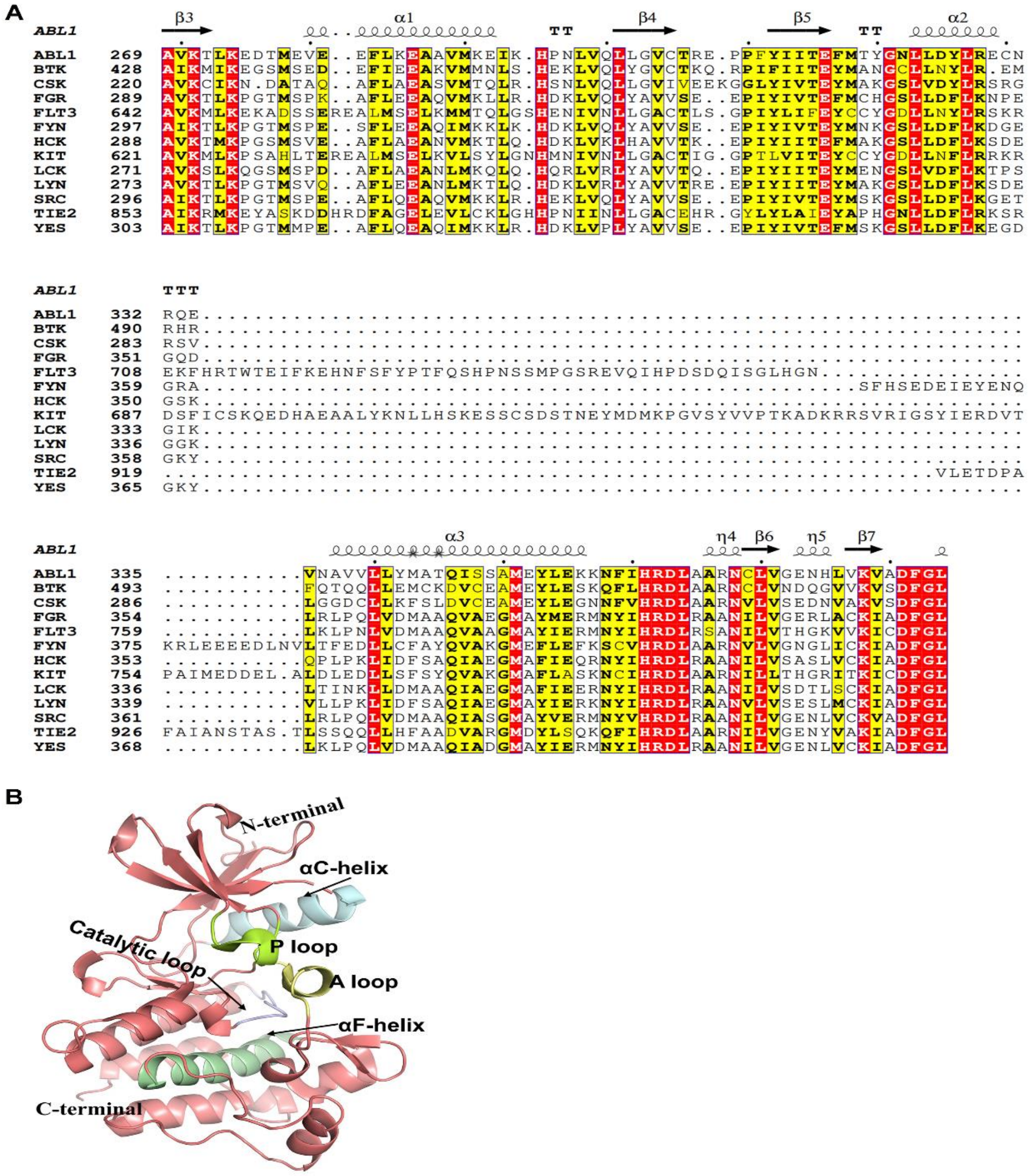

Figure S5. Supplementary data for Figure 4. (A) Sequence alignment is generated using ESPript 3.0 server. Secondary structure elements corresponding to ABL1's PDB code (3qri) are shown above the sequence. (B) The cartoon shows the structure of the ABL1 kinase domain. Key structural elements are colored in palecyan (αC-helix), limon (p-loop), paleyellow (A-loop), palegreen (αF-helix), lightblue (catalytic loop).

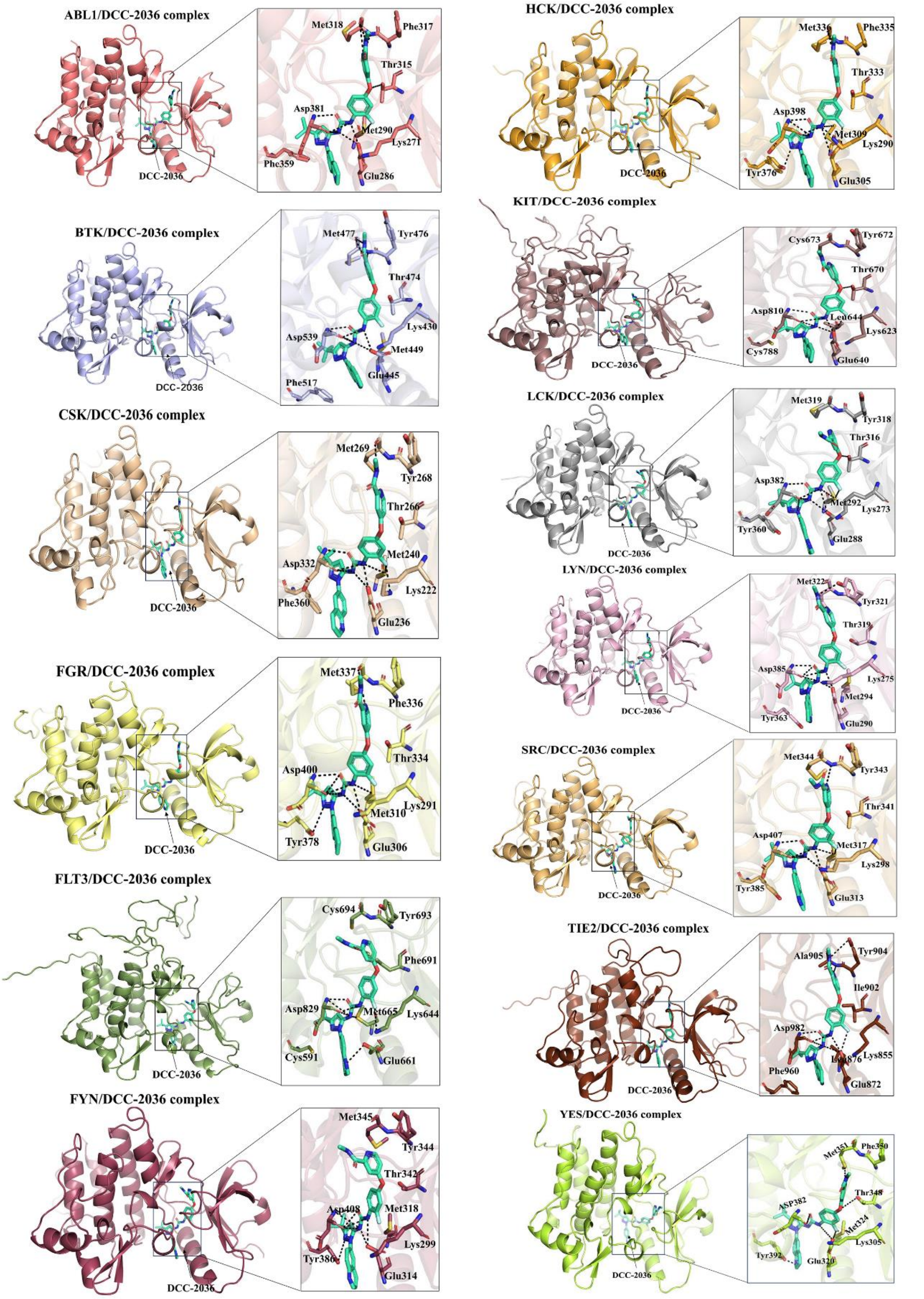

Figure S6. Supplementary data for Figure 4. Cartoon representation of the protein kinase for which structures in complex with DCC-2036 are known. Left: the overall complex structure generated by molecular docking; right: the main interactions between DCC-2036 and different kinases. Amino acids that produce hydrogen bonds are depicted in stick form, and the hydrogen bonds are indicated.

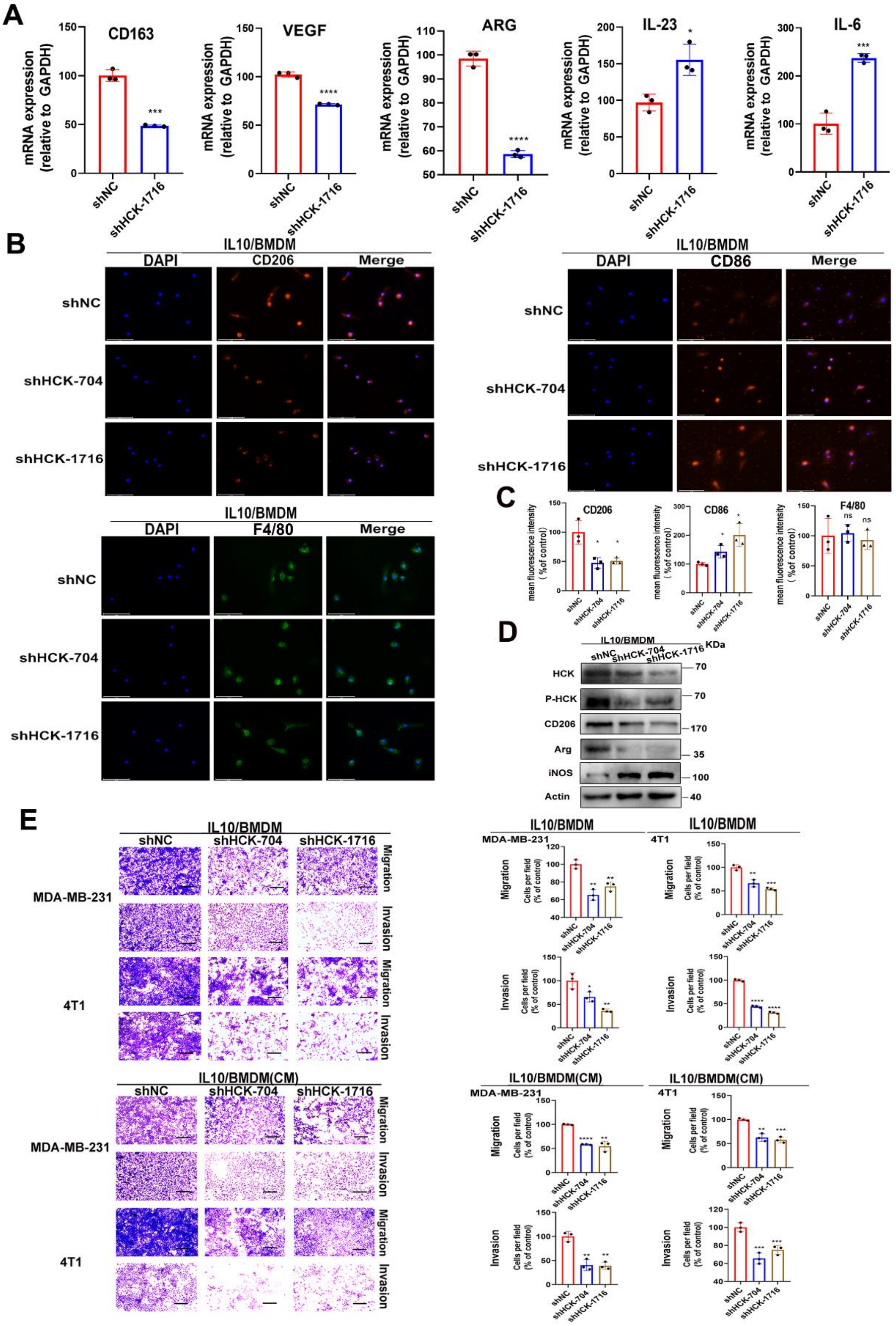

Figure S7. The impact of HCK knockdown on M2-polarized macrophages (IL10/BMDM) and its effect on migration and invasion of triple-negative breast cancer cells. (A) q PCR was used to detect the mRNA expression of M2 and M1 markers in IL10/BMDM after knocking down of HCK. (B) Immunofluorescence experiments were performed to assess the fluorescence expression of M2, M1, and total

macrophage markers in IL10/BMDM cells following HCK knockdown. Scale bars, 75 µm. (C) The histogram represents the mean value of three experiments. (D) Protein expression of M1 and M2 markers in IL10/BMDM cells with HCK knockdown. (E) Representative images of IL10/BMDM-treated with shHCK co-cultured with TNBC cells (MDA-MB-231 and 4T1 cells) were captured. Scale bars, 50 µm. The conditioned media generated by IL10/BMDM treated with shHCK were co-cultivated with TNBC cells. The statistical results were visually represented using a bar chart (right), displaying the Mean ± SD with n = 3. The significance of the findings was assessed through t-test analysis, *p<0.05， **p<0.01， ***p<0.001， ****p<0.0001.

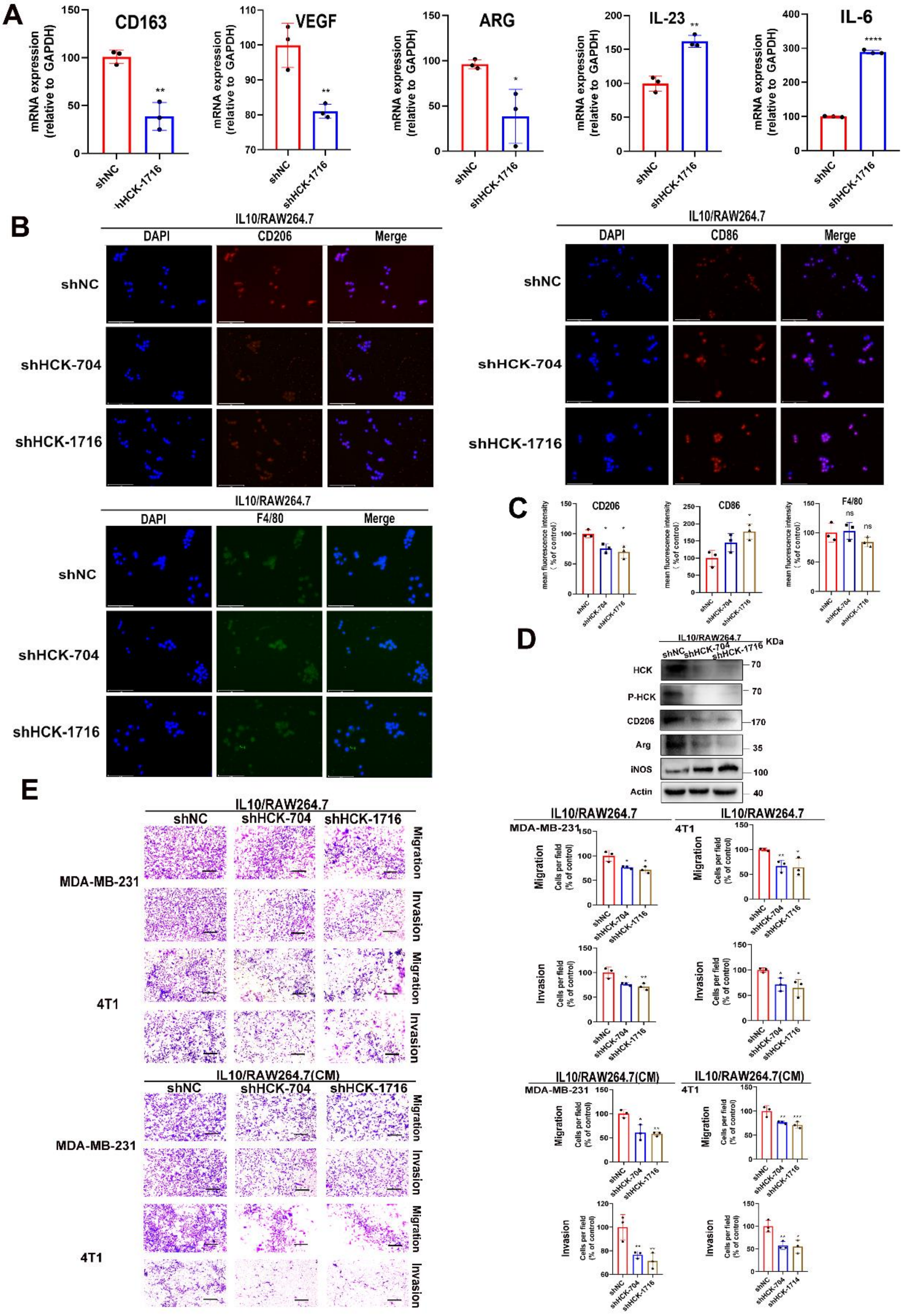

Figure S8. The impact of HCK knockdown on M2-polarized macrophages (IL10/ RAW264.7) and its effect on migration and invasion of triple-negative breast cancer cells. (A) q PCR was used to detect the mRNA expression of M2 and M1 markers in IL10/RAW264.7 after knocking down of HCK. (B) Immunofluorescence experiments were performed to assess the fluorescence expression of M2, M1, and total

macrophage markers in IL10/RAW264.7 cells following HCK knockdown. Scale bars, 75 μm. (C) The histogram represents the mean value of three experiments. (D) Protein expression of M1 and M2 markers in IL10/RAW264.7 cells with HCK knockdown. (E) Representative images of IL10/RAW264.7-treated with shHCK co-cultured with TNBC cells (MDA-MB-231 and 4T1 cells) were captured. Scale bars, 50 μm. The conditioned media generated by IL10/RAW264.7 treated with shHCK were co-cultivated with TNBC cells. The statistical results were visually represented using a bar chart(right), displaying the Mean ± SD with n = 3. The significance of the findings was assessed through t-test analysis, *$p<0.05$，**$p<0.01$，***$p<0.001$，****$p<0.0001$.

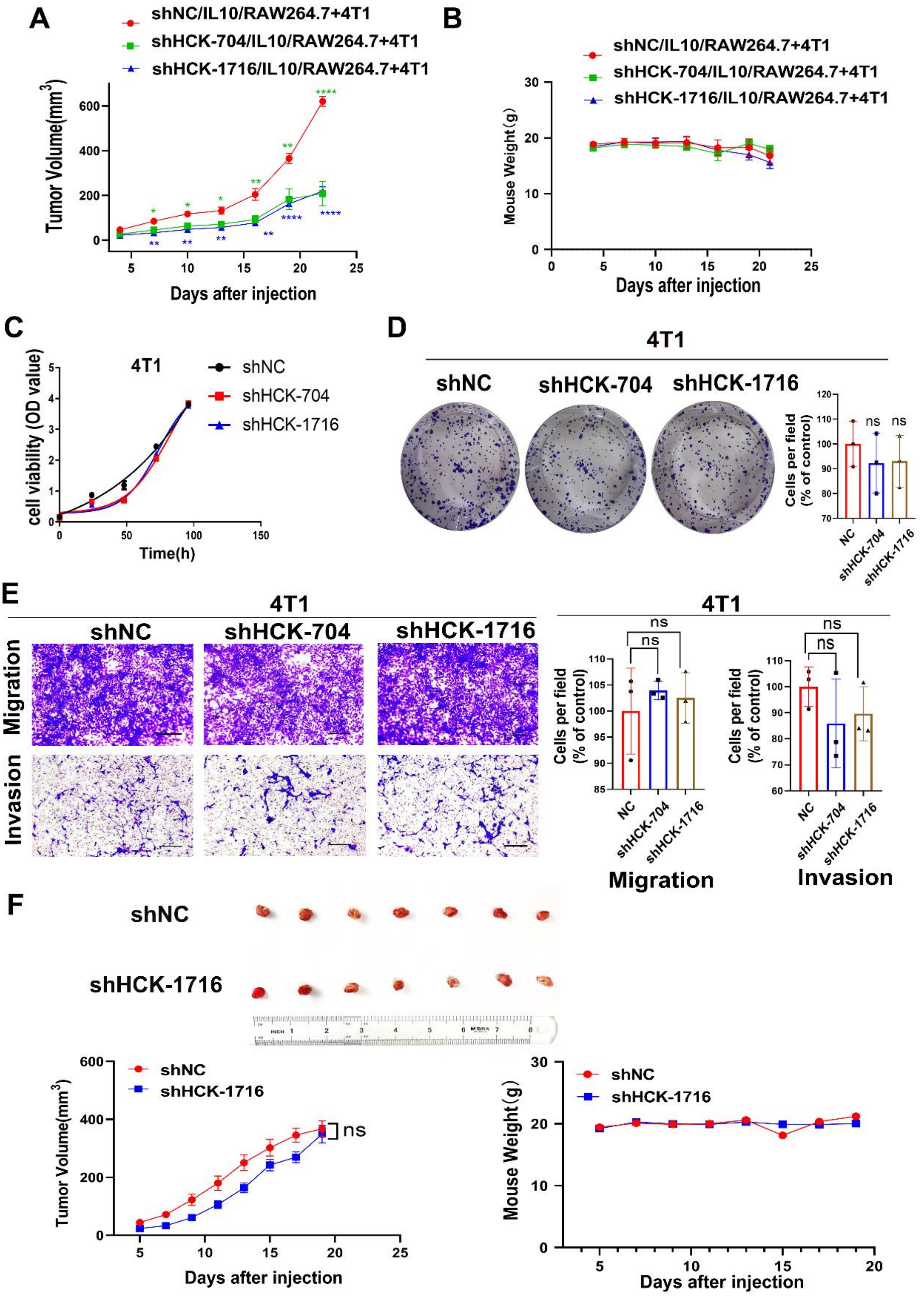

Figure S9. The impact of HCK knockdown in different cells on triple-negative breast cancer. (A) Mouse tumor growth curves were generated for the control, shHCK-704,and shHCK1716 (shNC/IL10/RAW264.7+4T1, shHCK704/IL10/RAW264.7+4T1, shHCK-704/IL10/RAW264.7+4T1), with a sample size of n=5 in each group. (B) Weight change curves of three groups of mice. (C) The CCK8 cell viability assay was performed to generate the cell growth curve for the NC group, shHCK-704 group, and

shHCK-1716 group in 4T1 cells. (D) By conducting a plate cloning experiment, the impact of HCK knockdown on the clonogenic ability of 4T1 cells was assessed. Cells transfected with shNC/shHCK-704/shHCK-1716 were seeded in a 6-well plate and after 7 days, the number of clones was quantified and presented in the accompanying graph (right). (E) To investigate the impact of HCK knockdown on cell migration and invasion ability, a total of 8×10^4 cells transfected with shNC/shHCK-704/shHCK-1716 were individually seeded in the upper chamber of Transwell inserts. Subsequently, the cells adhered to the polycarbonate membrane were captured and quantified using microscopy. Scale bars, 50 μm. (F) Representative images of tumor tissues from the control group and shHCK-1716 group with HCK knockdown, growth curves of tumors in mice from both groups, as well as weight change curves (n=7 for each group).

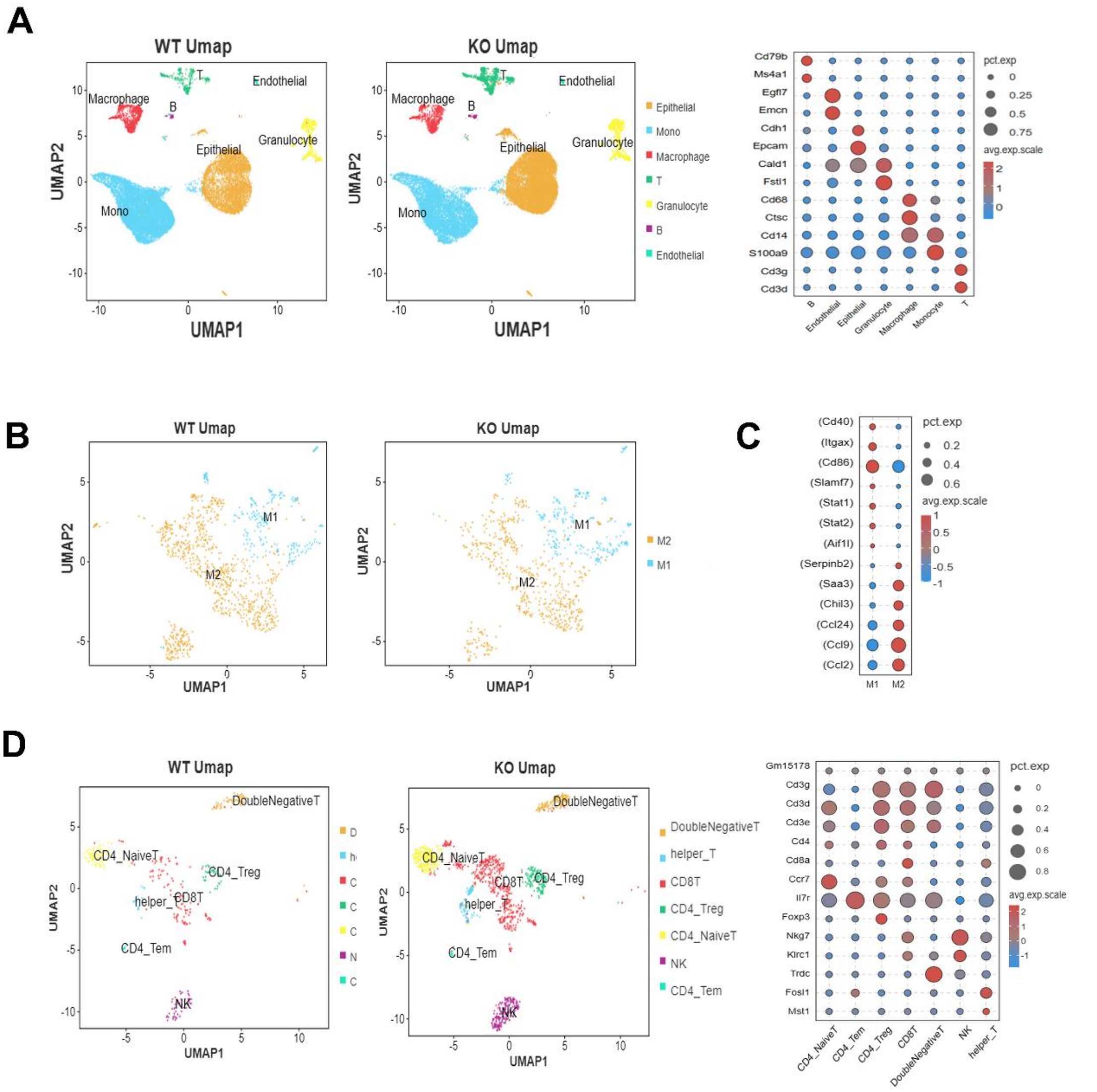

Figure S10. Supplementary data for Figure 5. (A) Umap plots illustrating the disparities between WT and HCK^{KO}, alongside bubble plots depicting the expression of marker genes for each cell type. (B) Umap plots depicting the distribution of M1 and M2 macrophages in wild-type (WT) and HCK^{KO} mice. (C) Analyze the gene dotplot of M1 and M2 macrophage markers in WT and HCK^{KO}. (D) UMAP plots depicting the overall distribution of distinct T cell subpopulations in both wild-type (WT) and HCK^{KO} samples; Dotplot illustrating marker gene expression specific to each T cell subpopulation.

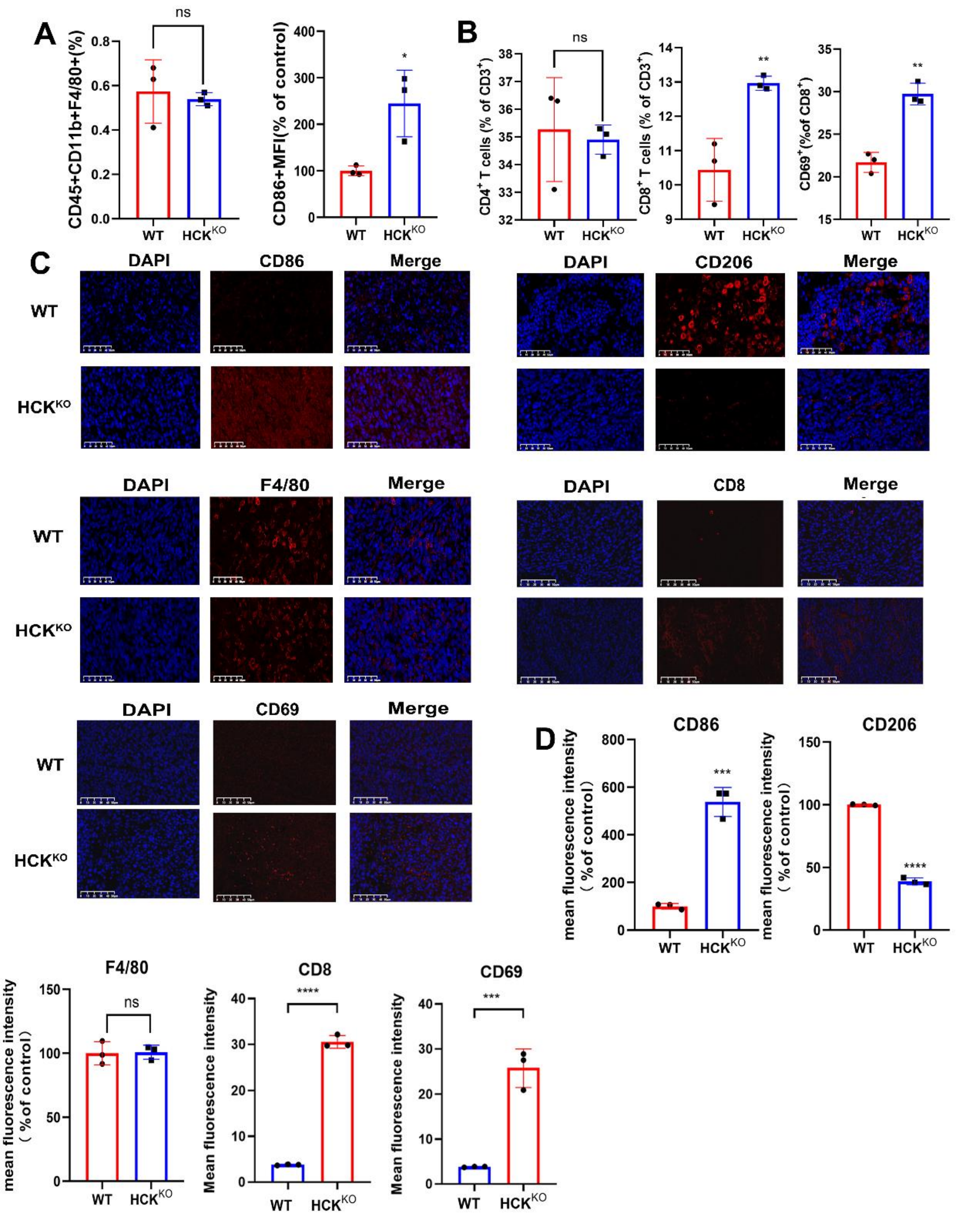

Figure S11. Supplementary data for Figure 5. (A) Flow cytometry experiments were conducted on tumor tissues obtained from the WT and HCK^{KO} mouse cohorts to quantify the total population of macrophages and assess the fluorescence intensity expression of M1 marker CD86 in both groups. (B) Using flow cytometry, we compared the CD4^{+} and CD8^{+} T cell numbers as well as CD69^{+} T cell numbers in tumor tissues from two groups of mice. (C) Immunofluorescence was employed to assess the expression levels of macrophage markers, quantify CD8^{+} T cell population, and evaluate activation marker of CD8^{+} T cells. The scale was set at 50 μm. (D) Immunofluorescence statistical analysis was performed based on three independent repeated experiments, and the results are presented as a bar chart with Mean ± SD. The

statistical significance was determined using t-test, where $*p<0.05$, $**p<0.01$, $***p<0.001$, $****p<0.0001$ indicate significant differences compared to control group while ns represents no significant difference.

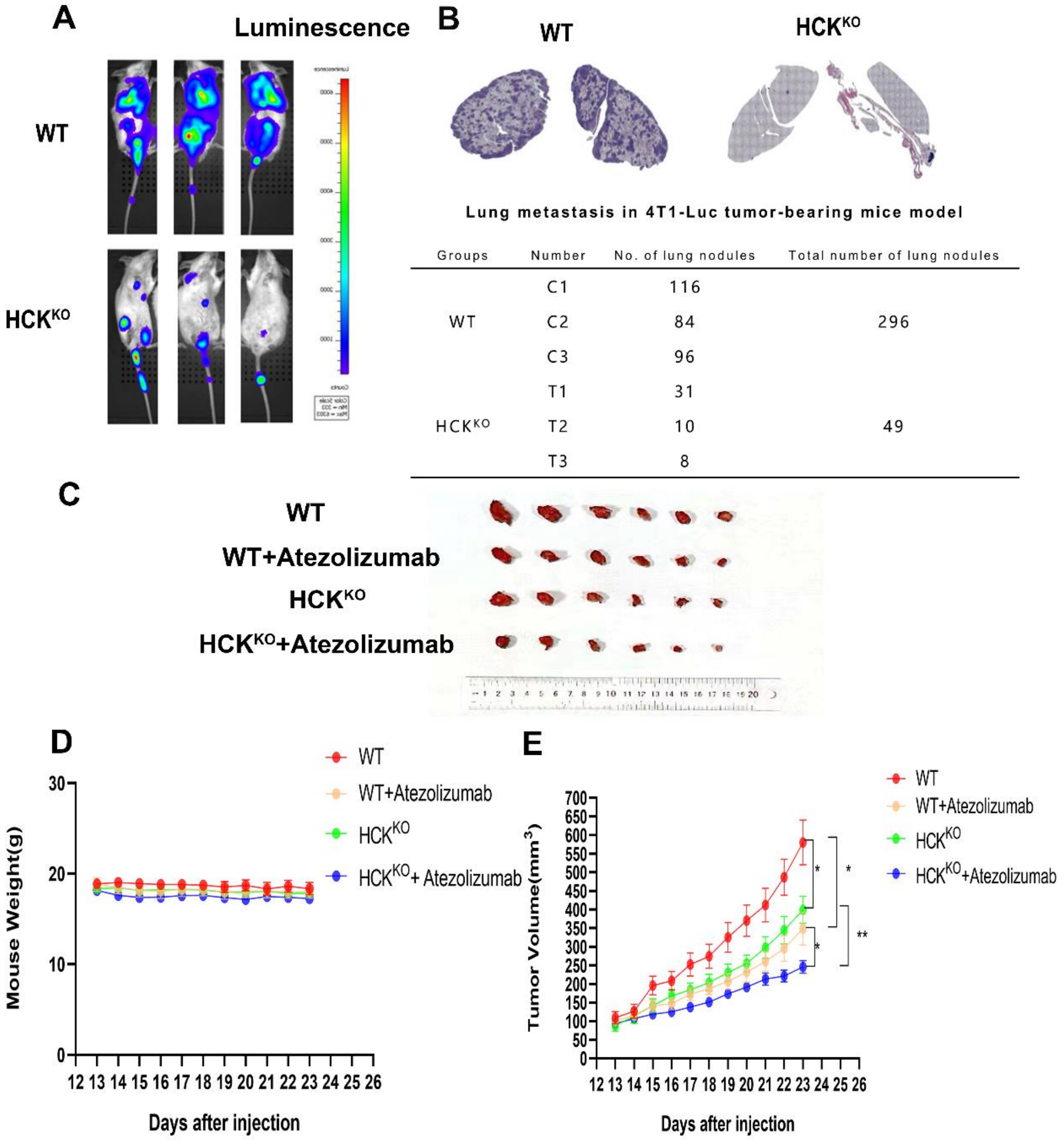

Lung metastasis in 4T1-Luc tumor-bearing mice model

Groups	Number	No. of lung nodules	Total number of lung nodules
	C1	116	
WT	C2	84	296
	C3	96	
	T1	31	
HCK^{KO}	T2	10	49
	T3	8	

Figure S12. HCK knockout suppressed TNBC metastasis in mice and enhanced the sensitivity of TNBC to PD-L1 inhibitor atezolizumab. (A) The live imaging of tumor metastasis was conducted in Balb/c mice, with the wild-type (WT) group depicted above and the HCK knockout (HCK^{KO}) group shown below (Obtain the image on the 30th day）. The sample size for both groups was n=3. (B) Relevant panoramic scans of HE-stained lung tissue sections from the WT group (upper left) and HCK^{KO} group (upper right) mice; Tabulated data presenting tumor nodule counts in lung tissues of both WT and HCK^{KO} groups (below). (C) The representative tumor graphs of four groups of mice were obtained in this study. Wild-type and HCK^{KO} Balb/c mice were inoculated with 2×10^6 4T1 cells in the fourth mammary fat pad. Upon reaching a tumor volume of 100-200 mm^3, the mice were randomly allocated into four groups: WT group; WT+Atezolizumab treatment group (5 mg/kg, administered via intraperitoneal injection); HCK^{KO} group; HCK^{KO}+Atezolizumab treatment group (administered using the same route as before). (D) Mouse weight curves of four groups. (E) Four groups of

mice's tumor growth curves. The significance of the findings was assessed through t-test analysis, *p<0.05， **p<0.01， ***p<0.001， ****p<0.0001.

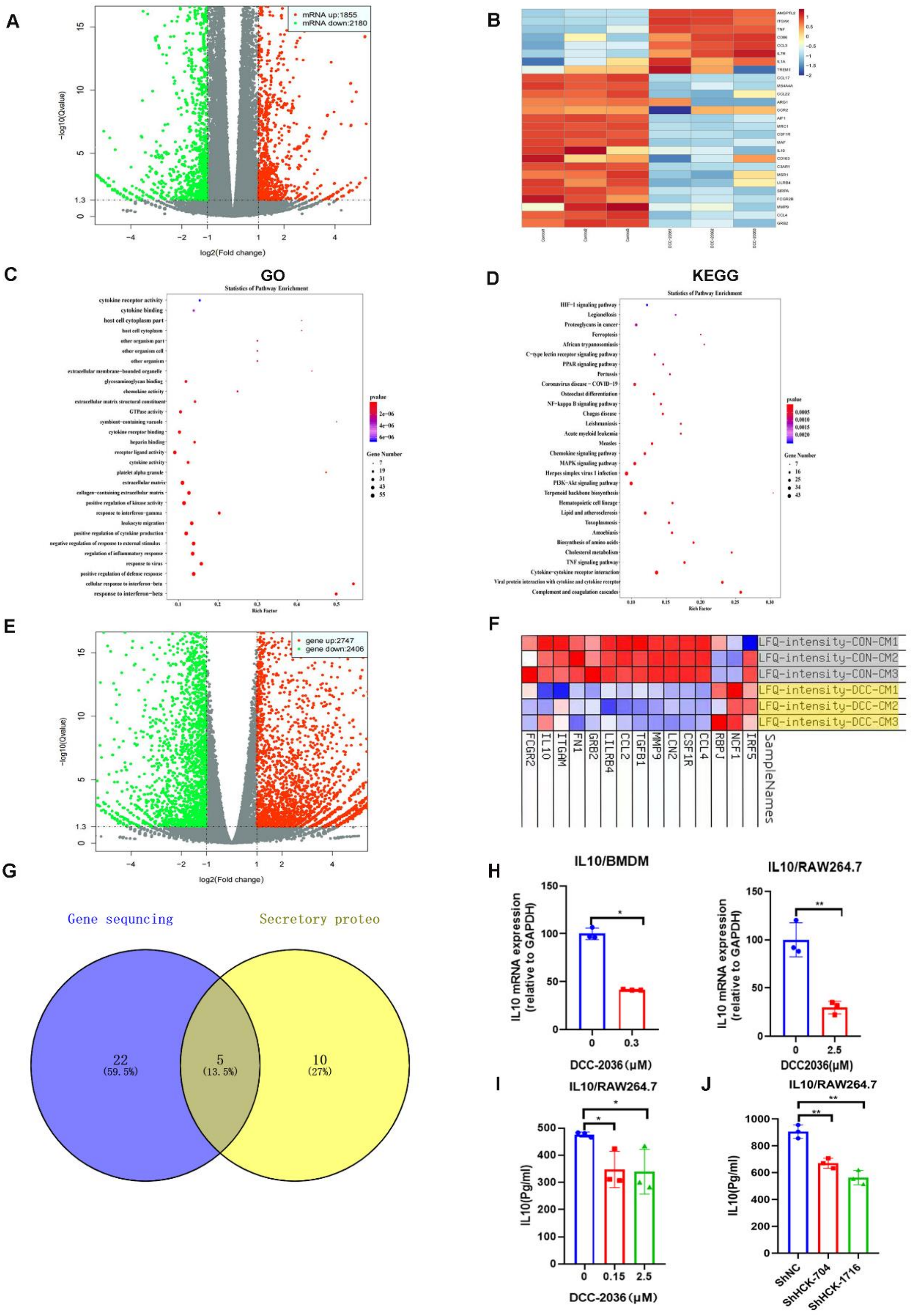

Figure S13. Related omics analysis of M2-type bone marrow-derived macrophages (BMDM) before and after DCC-2036 treatment. (A) Volcano plot illustrating the differential expression analysis of mRNA in M2-type BMDM transcriptomics before and after treatment with 0.3 μM DCC-2036. (B) Cluster heatmap depicting the expression patterns of differentially regulated M1 and M2 polarization markers in the transcriptome of M2-type BMDMs before and after treatment with 0.3 μM DCC-2036,

based on criteria of |log2(Fold Change)|>1 and P value<0.05. (C) GO bubble chart depicting transcriptomic analysis of M2-type BMDM before and after treatment with 0.3 μM DCC-2036. (D) KEGG enrichment bubble plot illustrating the transcriptomic analysis of M2-type BMDM before and after treatment with 0.3 μM DCC-2036. (E) Volcano plot depicting the proteomic analysis of secreted proteins in M2-type BMDM pre- and post-treatment with 0.3 μM DCC-2036. (F) A clustering diagram was generated to illustrate the differential expression of common macrophage M1 and M2 polarization markers in the secretome of M2-type BMDMs before and after treatment with 0.3 μM DCC-2036, based on criteria of |log2(Fold Change)|>1 and P value<0.05. (G) A Venn diagram was employed to analyze the intersection between changes in macrophage polarization indicators of transcriptome and secreted proteomic analysis, both before and after treatment with 0.3 μM DCC-2036. (H) The expression levels of IL10 mRNA in M2-type BMDM and RAW264.7 cells treated with DCC-2036 were assessed using q PCR analysis. (I) The concentration of IL10 in the culture medium of M2-type RAW264.7 treated with DCC-2036 was quantified using the ELISA method. (J) The concentration of IL10 in the culture medium of HCK knockdown M2- type RAW264.7 cells was quantified using the ELISA technique.

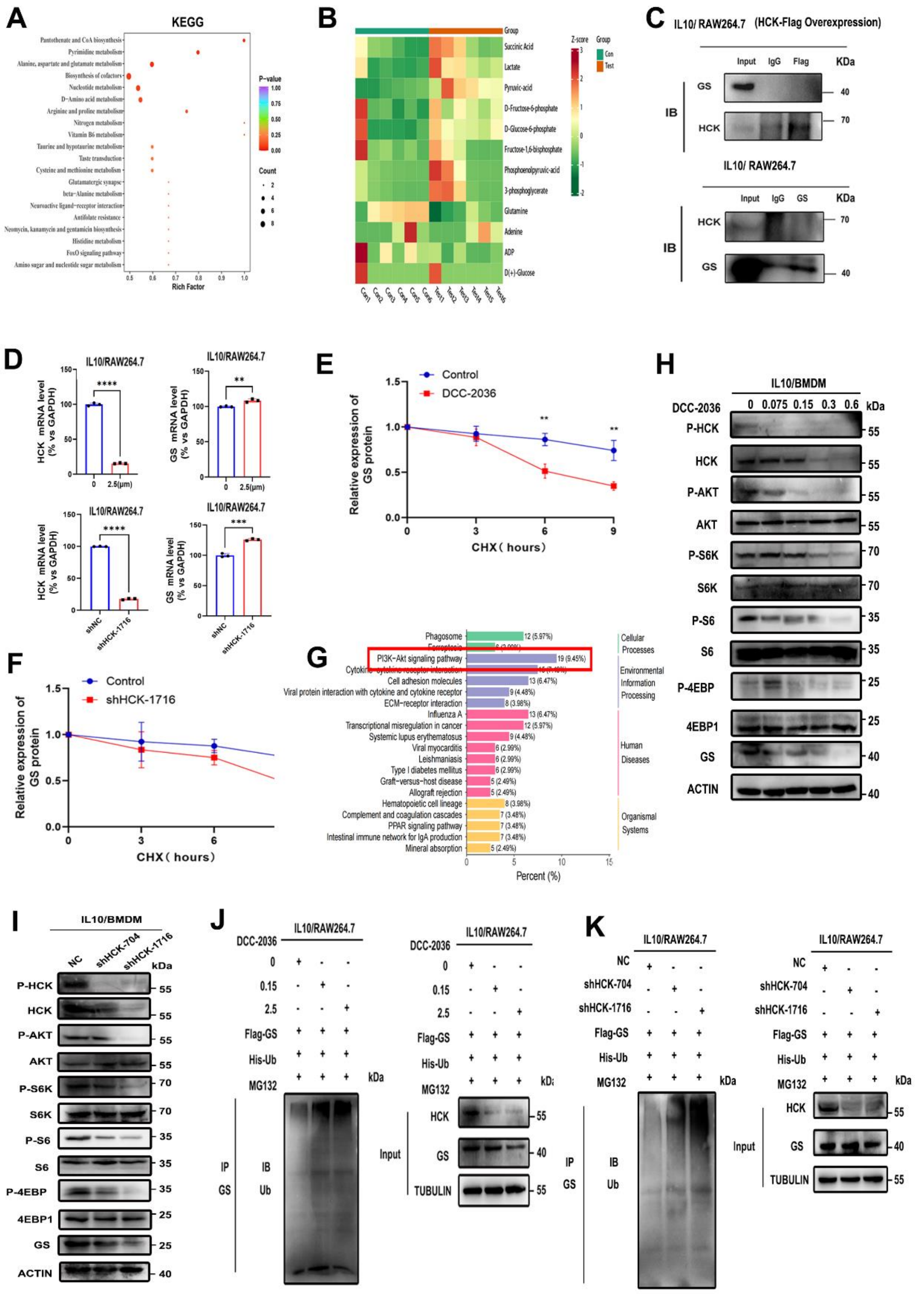

Figure S14. Supplementary data for Figure 6. (A) KEGG bubble plots of energy metabolism analysis of M2 BMDM before and after treatment with 0.3 μM DCC-2036; (B) Clustered heatmaps of energy metabolism analysis of M2 BMDM before and after treatment with 0.3 μM DCC-2036. (C) Lysates of IL10/RAW264.7 cells transfected with exogenous Flag-Hck plasmid and lysates of untransfected IL10/RAW264.7 cells were subjected to exogenous (top) and endogenous (bottom) Co-IP assays, respectively,

with Flag antibody for exogenous IP, GS antibody for endogenous IP, and anti-HCK, GS and Actin antibodies for IB detection of precipitates. IgG was the negative control and Input was the whole cell lysate. (D) q RT-PCR detection of HCK and GS mRNA expression in IL10/RAW264.7 after treatment with DCC-2036 (2.5 μM) or shHCK. (E-F) Statistical results for Figure 6F. Results are shown as bar graphs, mean ± s.d, n = 3 independent experiments. Statistical significance was determined by t-test. *$P<0.05$, **$P<0.01$. (G) KEGG analysis of transcriptomics showed that the differential genes regulated by HCK knockdown were mainly associated with the PI3K/AKT signaling pathway. (H) IL10/BMDM cells were treated with different concentrations of DCC-2036 for 48 h; cells were collected for Western Blotting to analyze the protein expression of P-HCK, HCK, P-AKT, AKT, mTOR-related markers and GS. (I) IL10/BMDM cells treated with shHCK were collected for Western Blotting to analyze protein expression of P-HCK, HCK, P-AKT, AKT, mTOR-related markers and GS. (J-K) Flag-tagged GS plasmid and His-tagged ubiquitin plasmid were co-transfected into IL10/ RAW264.7 cells with or without DCC-2036/knockdown of HCK. The cells were treated with the proteasome inhibitor, MG132 (20 μM) for 8 h, and then GS ubiquitination was assessed.

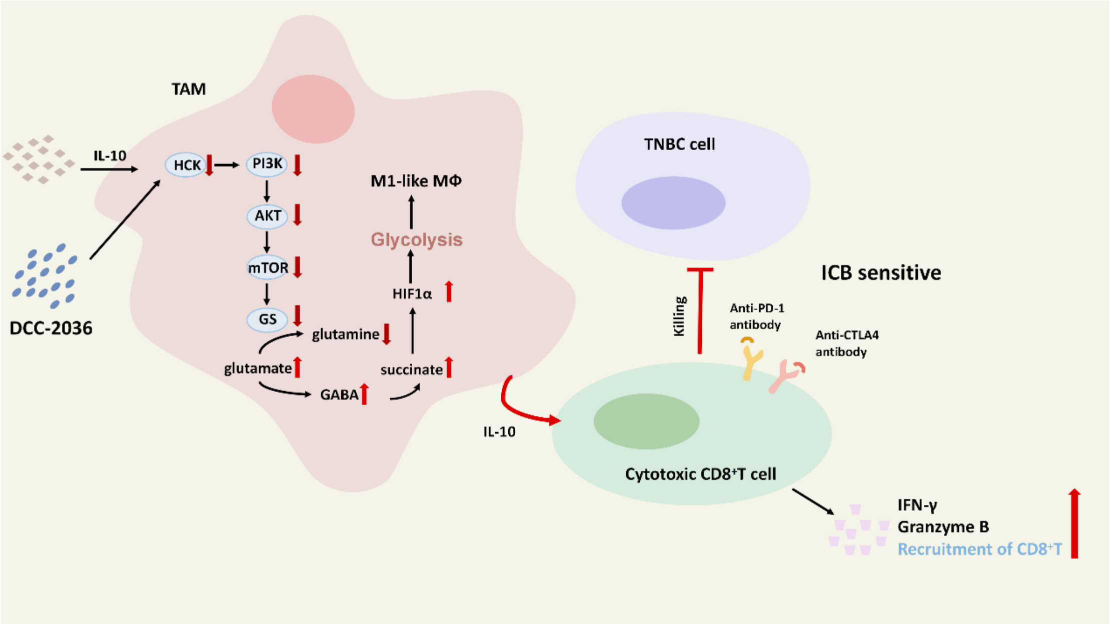

Figure S15. Schematic representations of the immune activation effect of DCC-2036 in triple-negative breast cancer. DCC-2036 induces repolarization of TAMs from M2 to M1 type and enhances anti-tumor CD8^{+} T cell immunity in TNBC. Mechanistically, targeting inhibition of HCK in TAMs regulates the downstream PI3K/AKT-mTOR-GS-HIF1α signaling pathway, leading to a reprogramming of TAM metabolism from oxidative phosphorylation to glycolysis. This metabolic shift repolarizes TAMs to the M1 phenotype, resulting in a decrease in IL-10 secretion, which enhances the immune response of anti-tumor CD8^{+} T cells and increases the sensitivity of TNBC to immune checkpoint blockade therapy.

Table S1. Primer sequences for qRT PCR

Gene	Orientations	Primer sequence（5’-3’）
IL-23	Forward	AGCGGGACATATGAATCTACTAAGAGA
	Reverse	GTCCTAGTAGGGAGGTGTGAAGTTG
IL-6	Forward	GAAACCGCTATGAAGTTCCTCTCTG
	Reverse	GTATCCTCTGTGAAGTCTCCTCTCC
CD163	Forward	GTTTGTGGAGCCATTCTATTGG
	Reverse	GGAAACTGTAAGTCGCTGAATC
VEGF	Forward	CAACTTCTGGGCTCTTCTCG
	Reverse	CCTCTCCTCTTCCTTCTCTTCC
ARG	Forward	CATATCTGCCAAAGACATCGTG
	Reverse	GACATCAAAGCTCAGGTGAATC
iNOS	Forward	ACTCAGCCAAGCCCTCACCTAC
	Reverse	TCCAATCTCTGCCTATCCGTCTCG
CCL5	Forward	GTATTTCTACACCAGCAGCAAG
	Reverse	TCTTGAACCCACTTCTTCTCTG
IL-10	Forward	AGCCTTATCGGAAATGATCCAGT
	Reverse	GGCCTTGTAGACACCTTGGT

Table S2 Summary of ADMET analysis results for compound DCC-2036

Pharmacokinetics/Type	Value
**1. Physicochemical Property**	
Molecular Weight (MW)	553.22
Volume	554.522
Density	0.998
nHA	10
nHD	3
nRot	10
nRing	5
MaxRing	10
nHet	11
fChar	0
nRig	30
Flexibility	0.333
Stereo Centers	0
TPSA	123.06
logS	-6.024
logP	4.489
logD7.4	3.754
pka (Acid)	9.637
pka (Base)	4.131
Melting point	176.485
Boiling point	362.704
**2. Absorption**	
Caco-2 Permeability	-5.042
MDCK Permeability	0
PAMPA	--
Pgp inhibitor	+++
Pgp substrate	---
HIA	---
F20%	---
F30%	+
F50%	+++
**3. Distribution**	
PPB	0.988
VDss	1.925
BBB	---
Fu	0.01
OATP1B1 inhibitor	+++
OATP1B3 inhibitor	+++
BCRP inhibitor	---

Pharmacokinetics/Type	Value
**5. Excretion**	
CLplasma	2.144
T1/2	1.358
**6. Toxicity**	
hERG Blockers	0.591
hERG Blockers (10um)	0.17
DILI	1
AMES Toxicity	0.827
Rat Oral Acute Toxicity	0.277
FDAMDD	0.734
Skin Sensitization	0.01
Carcinogenicity	0.618
Eye Corrosion	0
Eye Irritation	0.015
Respiratory	0.671
Human Hepatotoxicity	0.933
Drug-induced Nephrotoxicity	0.996
Drug-induced Neurotoxicity	0.967
Ototoxicity	0.932
Hematotoxicity	0.868
Genotoxicity	1
RPMI-8226 Immunitoxicity	0.155
A549 Cytotoxicity	0.216
Hek293 Cytotoxicity	0.834
BCF	1.039
IGC50	3.654
LC50DM	5.773
LC50FM	4.784
**7. Toxicophore Rules**	
Aquatic Toxicity Rule	1
Genotoxic Carcinogenicity Mutagenicity Rule	1
NonGenotoxic Carcinogenicity Rule	1
Skin Sensitization Rule	4
Acute Toxicity Rule	0
NonBiodegradable	5
SureChEMBL Rule	0
FAF-Drugs4 Rule	1
**8. Tox21 Pathway**	
NR-AhR	+++

MRP1 inhibitor	++
BSEP inhibitor	+++

4. Medicinal Chemistry

QED	0.235
SAscore	Easy
GASA	Easy
Fsp3	0.167
MCE-18	31
NPscore	-1.849
Lipinski Rule	Accepted
Pfizer Rule	Accepted
GSK Rule	Rejected
GoldenTriangle	Rejected
PAINS	0
Alarm_NMR Rule	1
BMS Rule	0
Chelating Rule	0
Colloidal aggregators	0.928
FLuc inhibitors	0.816
Blue fluorescence	0.223
Green fluorescence	0.951
Reactive compounds	0.004
Promiscuous compounds	0.791

NR-AR	---
NR-AR-LBD	---
NR-Aromatase	---
NR-ER	---
NR-ER-LBD	---
NR-PPAR-gamma	---
SR-ARE	+++
SR-ATAD5	--
SR-HSE	---
SR-MMP	+++
SR-p53	-

*For the classification endpoints , the prediction probability values are transformed into six symbols: 0-0.1 (---), 0.1-0.3 (--), 0.3-0.5 (-), 0.5-0.7 (+), 0.7-0.9 (++), and 0.9-1.0 (+++).

**The corresponding relationships of the three labels are as follows: green: excellent; yellow: medium; red: poor.

Table S3 Prediction of CYP substrates for compound DCC-2036

CYP Member	Prediction
CYP1A2	Non-substrate
CYP2A6	Non-substrate
CYP2B6	Non-substrate
CYP2C8	Non-substrate
CYP2C9	Non-substrate
CYP2C19	Substrate
CYP2D6	Non-substrate
CYP2E1	Non-substrate
CYP3A4	Substrate

Table S4 Prediction of SULT substrates for compound DCC-2036

SULT Member	Prediction
SULT1A1/2/3/4	Non-substrate
SULT1B1	Non-substrate
SULT1C2	Non-substrate
SULT1C4	Non-substrate
SULT1E1	Non-substrate
SULT2A1	Non-substrate
SULT2B1a/b	Non-substrate

Table S5 Main metabolic reactions and their priorities for DCC-2036

Rank	Priority Score	Reaction Type
1	0.276	N-oxidation_(-N=)
1	0.276	Aromatic_hydroxylation_(meta_to_carbon)
1	0.276	Aromatic_hydroxylation_(ortho_to_2_substituents)
1	0.276	N-acetylation_(NH1-CH3)
1	0.276	Aromatic_hydroxylation_(para_to_carbon)
6	0.236	Hydrolysis_(secondary_amide)
6	0.236	N-demethylation_(R-NHCH3)
6	0.236	Amine hydroxylation
9	0.212	Aromatic_hydroxylation_(para_to_carbon)
9	0.212	Aromatic_hydroxylation_(para_to_carbon)
9	0.212	N-oxidation_(-N=)
9	0.212	Aromatic_hydroxylation_(ortho_to_nitrogen)
13	0.164	Carboxylation_(primary_carbon_next_to_quart_carbon)
13	0.164	Aliphatic_hydroxylation_(primary_carbon_next_to_quart_carbon)
15	0.12	Aliphatic hydroxylation
16	0.112	Aromatic hydroxylation
17	0.104	Hydrolysis_(urea_or_carbonate)
17	0.104	Amine hydroxylation
17	0.104	Hydrolysis_(urea_or_carbonate)
17	0.104	Amine hydroxylation
17	0.104	Hydrolysis_(urea_or_carbonate)
17	0.104	Hydrolysis_(urea_or_carbonate)
23	0.096	Aromatic_hydroxylation_(ortho_to_2_substituents)
23	0.096	N-oxidation
25	0.088	N-glucuronidation_(aromatic_=n-)
26	0.068	N-glucuronidation_(aniline_NH1-R)
27	0.064	Aromatic_hydroxylation_(para_to_carbon)
28	0.06	Aromatic hydroxylation
28	0.06	N-oxidation
30	0.056	Aromatic_hydroxylation_(ortho_to_nitrogen)
31	0.052	N-glucuronidation_(aniline_NH1-R)
31	0.052	Aromatic_hydroxylation_(ortho_to_oxygen)
31	0.052	Aromatic_hydroxylation_(ortho_to_oxygen)
34	0.04	N-glucuronidation_(aromatic_=n-)
35	0.024	Dehydration of N-C bond
36	0.021	Carbamide cleavage
36	0.021	Carbamide cleavage
38	0.016	N-glucuronidation_(aromatic_=n-)
39	0.012	Oxidative dehalogenation benzyl
40	0.01	O-dearylation
40	0.01	O-dearylation

Table S6 Molecular docking scores of different protein tyrosine kinases bounding to DCC-2036

Protein Kinases	Binding Free Energy(kcal/mol)
ABL1	-13.7
BTK	-12.3
CSK	-10.4
FGR	-12.7
FLT3	-8.4
FYN	-10.7
HCK	-12.2
KIT	-9.2
LCK	-12.3
LYN	-12.1
SRC	-12.1
TIE2	-10.3
YES	-12.5
